# The acquisition of clinically relevant amoxicillin resistance in *Streptococcus pneumoniae* requires ordered horizontal gene transfer of four loci

**DOI:** 10.1371/journal.ppat.1010727

**Published:** 2022-07-25

**Authors:** Paddy S. Gibson, Evan Bexkens, Sylvia Zuber, Lauren A. Cowley, Jan-Willem Veening

**Affiliations:** 1 Department of Fundamental Microbiology, Faculty of Biology and Medicine, University of Lausanne, Lausanne, Switzerland; 2 Department of Biology & Biochemistry, Milner Centre for Evolution, University of Bath, Bath, United Kingdom; Boston Children’s Hospital, UNITED STATES

## Abstract

Understanding how antimicrobial resistance spreads is critical for optimal application of new treatments. In the naturally competent human pathogen *Streptococcus pneumoniae*, resistance to β-lactam antibiotics is mediated by recombination events in genes encoding the target proteins, resulting in reduced drug binding affinity. However, for the front-line antibiotic amoxicillin, the exact mechanism of resistance still needs to be elucidated. Through successive rounds of transformation with genomic DNA from a clinically resistant isolate, we followed amoxicillin resistance development. Using whole genome sequencing, we showed that multiple recombination events occurred at different loci during one round of transformation. We found examples of non-contiguous recombination, and demonstrated that this could occur either through multiple D-loop formation from one donor DNA molecule, or by the integration of multiple DNA fragments. We also show that the final minimum inhibitory concentration (MIC) differs depending on recipient genome, explained by differences in the extent of recombination at key loci. Finally, through back transformations of mutant alleles and fluorescently labelled penicillin (bocillin-FL) binding assays, we confirm that *pbp1a*, *pbp2b*, *pbp2x*, and *murM* are the main resistance determinants for amoxicillin resistance, and that the order of allele uptake is important for successful resistance evolution. We conclude that recombination events are complex, and that this complexity contributes to the highly diverse genotypes of amoxicillin-resistant pneumococcal isolates.

## Author summary

*Streptococcus pneumoniae* (the pneumococcus) is an asymptomatic coloniser of the human nasopharynx and a common cause of middle ear infections and pneumonia. The frontline treatment for bacterial respiratory tract infections is amoxicillin, a β-lactam antibiotic that inhibits cell wall synthesis. The pneumococcus is naturally competent and β-lactam resistance in this species is acquired by recombination of large stretches of foreign DNA into genes encoding the target penicillin binding proteins, resulting in reduced drug binding affinity. For amoxicillin, the exact mechanism of resistance development is not well understood. Here, we sequentially transformed genomic DNA from a resistant clinical isolate into two different susceptible strains and selected on amoxicillin. This resulted in a large collection of strains with varying amoxicillin resistances and a known pedigree. Using whole genome sequencing and fluorescently labelled penicillin binding assays, we were able to confirm that alterations to penicillin binding proteins Pbp2x, Pbp2b, and Pbp1a, as well as another cell wall synthesis protein MurM are essential for amoxicillin resistance. We also show that the resistant alleles must be acquired in a specific order for optimal resistance evolution, and that the inherent complexity of recombination itself may contribute to the enormous diversity of resistant pneumococcal isolates.

## Introduction

Genomic plasticity through frequent and large-scale recombination drives the evolution of antibiotic resistance in *Streptococcus pneumoniae* (the pneumococcus) [1]. This naturally competent member of the human nasopharyngeal commensal microbiota opportunistically causes otitis media as well as severe invasive diseases such as pneumonia, bacteremia, and meningitis [2,3]. Despite conjugate vaccine introduction, the species remains an important human pathogen, as vaccine-escape and antibiotic resistant variants constantly arise [4,5].

Variants occur primarily through natural transformation and homologous recombination of DNA from strains or closely related species that occupy the same niche, such as *S*. *mitis* and *S*. *oralis* [6–8]. One case study showed more than 7% of the genome had been transferred between two pneumococcal strains during the polyclonal infection of a pediatric patient over three months [9]. Early studies of transformation in the pneumococcus showed uptake of fragments ranging from 2–6 Kb [10], but cell-to-cell contact was found to facilitate transfer of long DNA fragments [11], and recombinant regions as large as 30–50 Kb in length have been observed in clinically relevant lineages such as PMEN-1 [4,5,12]. Events such as these have played a significant role in shaping the pneumococcal pangenome [13].

**Fig 1:**
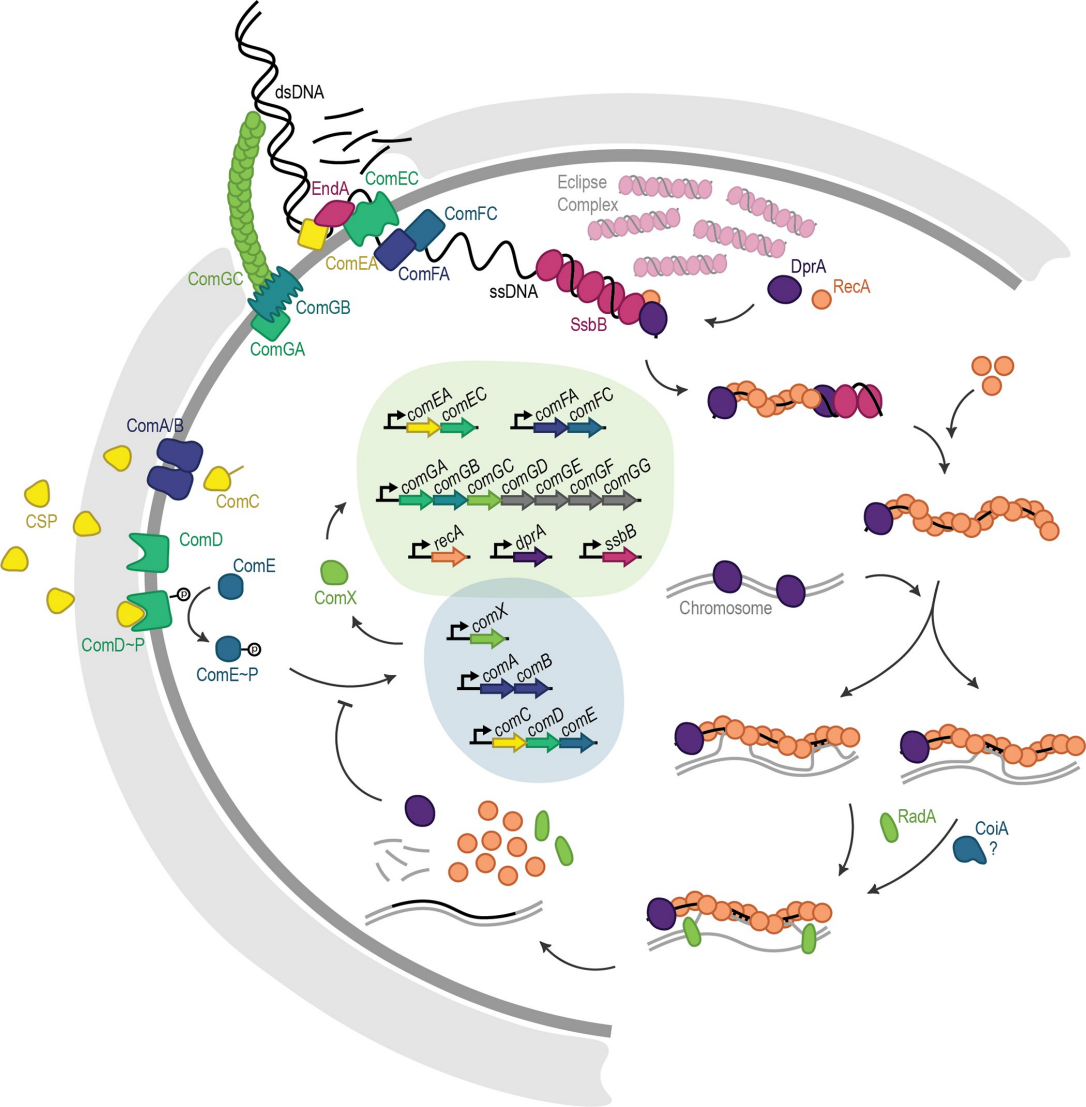
DNA uptake and recombination during natural competence in *Streptococcus pneumoniae*. Schematic overview of the regulatory steps required for the pneumococcus to uptake and recombine extracellular DNA. Competence is activated when CSP interacts with the ComDE two-component system, where phosphorylated ComE activates transcription of ComX and other early competence genes (darker grey bubble). This results in a positive feedback loop, where ComC is produced, then modified and exported from the cell by ComA/B as CSP. Meanwhile, ComX activates transcription of late competence genes, including the type IV-like pilus (ComGC), which brings DNA within reach of endonuclease EndA. EndA performs random nicks, then degrades one strand as the other is pulled into the cell and associated with DprA, RecA, and SsbB, to protect from degradation (exact mechanism not fully understood). DprA mediates loading of RecA onto SsbB-ssDNA eclipse complexes to form the presynaptic filament which then searches the chromosome for homology. RecA-ssDNA filaments may interact with the chromosome at multiple points, while successful base pairing between incoming and chromosomal DNA facilitates strand invasion and exchange. The helicase RadA facilitates D-loop extension, and CoiA is essential for successful recombination during transformation, although its exact role is not known. DprA is also involved in competence shut off, through blocking phosphorylation of ComE.

Natural competence is activated when the competence stimulating peptide (CSP) interacts with the ComDE two-component system [14] (Fig 1), where phosphorylated ComE activates transcription of the genes encoding the alternative sigma factor *comX* [15–17]. ComX then activates transcription of the late *com* genes necessary for DNA uptake and recombination. This includes the type IV-like pilus (ComGC), endonuclease EndA, and transport proteins ComFA, and ComEC [18–22]. RecA and the transformation-dedicated DNA processing protein A (DprA) are essential for single-stranded DNA (ssDNA) uptake without degradation [23]. Although not fully understood, DprA and RecA are thought to polymerize on ssDNA upon cell entry, initiating the homology search, while additional DNA molecules are then bound by multiple SsbB proteins protecting them from degradation by endogenous nucleases [23,24]. It has been hypothesized that the accumulation of stable ssDNA-SsbB eclipse complexes in the cytoplasm enables successive recombination events during one competence window, increasing genetic plasticity [23]. Sequestered ssDNA can then be accessed by DprA, which mediates the loading of RecA onto the pre-synaptic filament [25–27]. DprA is also involved in competence shut-off, along with ComX and RpoD sigma factor competition, and CSP degradation by HtrA [16,28–31].

Competence is tightly controlled, with expression of most genes turned off within 20 minutes of CSP induction, providing a small window for DNA uptake and recombination [32]. To accelerate homology detection, the RecA-ssDNA presynaptic filament has been shown to interact with the chromosome in 3-dimensional space at multiple points of contact [33–35]. Displacement-loop (D-loop) formation is facilitated by base pairing between the chromosome and the ssDNA molecule, within the two DNA-binding sites of RecA [36,37]. Eight consecutive bases must be successfully paired before the DNA-DNA interaction is stabilized and strand exchange can occur [38,39]. In *S*. *pneumoniae*, D-loop extension is promoted by RadA (DnaB-type helicase) which helps to unwind chromosomal DNA, extending recombination over long genomic distances [40], and while its function is not known, CoiA is essential for successful recombination [26,41]. Although initial testing is highly stringent, once crossover has started and the region of complementation is extended, stringency decreases, allowing some mismatches and thereby increasing desirable genetic variation [42,43]. This is aided by the generalized mismatch repair system in *S*. *pneumoniae* (hex) which is not induced during competence [44], is only able to identify mismatches in the donor-recipient heteroduplex before D-loop resolution [44,45], [45] and is easily overwhelmed by excess mismatches [46].

Penicillin binding proteins (PBPs) are key β-lactam resistance determinants and are essential for bacterial cell wall synthesis by catalyzing the transglycosylation (TG) and transpeptidation (TP) reactions responsible for peptidoglycan (PGN) formation [47]. While highly conserved in penicillin (PEN) sensitive strains, PBPs of resistant isolates are highly variable, with up to 10% of amino acid changes [48]. Significant amounts of this variation was recently confirmed to be acquired from *S*. *mitis* [49]. These large-scale changes alter the structure of the active sites, reducing β-lactam binding affinity [48]. Amoxicillin (AMX) is the first line oral antibiotic prescribed for bacterial lower respiratory tract infections. AMX resistant strains first appeared in the mid-90s in the United States and France [50,51] and are currently on the rise in Spain, a phenomenon which correlates with increased usage of oral AMX/clavulanic acid [52]. Early AMX resistant lineages were closely related to known PEN resistance clones already circulating in the population [51,53]. Sequencing of *pbp* alleles from susceptible and resistant strains found high variation in *pbp2x* and *pbp1a*, while SNPs shared between AMX resistant isolates were only found in *pbp2b* [54–57]. However, no SNP or block of SNPs in any one *pbp*, when transferred to a susceptible strain, has been sufficient to confer resistance in a susceptible strain [55,58]. This is not entirely surprising, as the transpeptidase activity of PBPs is an essential function, and modifications to the active site are likely to have deleterious effects which need to be compensated for [59]. One mechanism of fitness compensation appears to be through substitutions in MurM, which results in abnormally high proportions of branched muropeptides in the cell wall [60–63]. Branched muropeptides were shown to have increased importance in strains depleted for Pbp2b, indicating a link between muropeptide composition and cell elongation [64]. Other non-target proteins which have been implicated in resistance include CpoA (LafB) [65] and CiaH [66].

Although multiple residues in Pbp1a, Pbp2x, and Pbp2b were found to be under positive selection in AMX resistant isolates [67], a specific a block of ten substitutions in the 590–641 region of the Pbp2b TP domain is repeatedly associated with AMX resistance [54–56]. However, transformation of *pbp2x*, *pbp2b*, and *pbp1a* alleles to AMX susceptible strain R6 did not recreate the AMX resistance of the donor strain, and the addition of *murM* had no effect. Only when genomic DNA was used could the original resistance level be achieved, indicating the role of a non-*pbp* or *murM* resistance determinant [56,57], or unknown epistatic interactions [68]. Interestingly, other studies have found a correlation between *murM* mutations and AMX resistance, although their presence depended on the *pbp2b* allele [52,54]. Taken together, these studies strongly suggest the existence of multiple routes to AMX resistance and imply a tight link in the roles of Pbp2b and MurM in the mechanisms.

To study recombination and the development of AMX resistance in the pneumococcus in more detail and identify possible evolutionary routes towards AMX resistance, we sequentially transformed genomic DNA from a resistant clinical isolate (serotype 11A, ST6521, German National Reference Centre for Streptococci 2017, SN75752) into two different recipient strains coming from different clonal complexes (serotype 2 D39V and serotype 4 TIGR4). Whole genome sequencing showed recipient-dependent, genome-wide mutation uptake. In addition to identifying the necessary determinants for AMX resistance, we found a strong tendency towards the order of *pbp* and *murM* allele uptake. Long read Pacbio sequencing excluded a role for DNA methylation or genomic rearrangements or movement of genetic mobile elements as resistance determinants. We also investigated non-contiguous recombination, previously observed in *S*. *pneumoniae*, *Haemophilus influenzae*, and *Helicobacter pylori* [8,69,70], and propose a model for how these complex events occur. To conclude, we show that the uptake of four alleles from a resistant strain (*pbp2x*, *pbp2b*, *pbp1A* and *murM*) is required and sufficient for clinically relevant amoxicillin resistance development in sensitive *S*. *pneumoniae*, and that necessary fitness compensation may define the order of allele uptake.

## Results

### Serial transformation with genomic DNA from an AMX resistant strain increased AMX MIC

**Fig 2:**
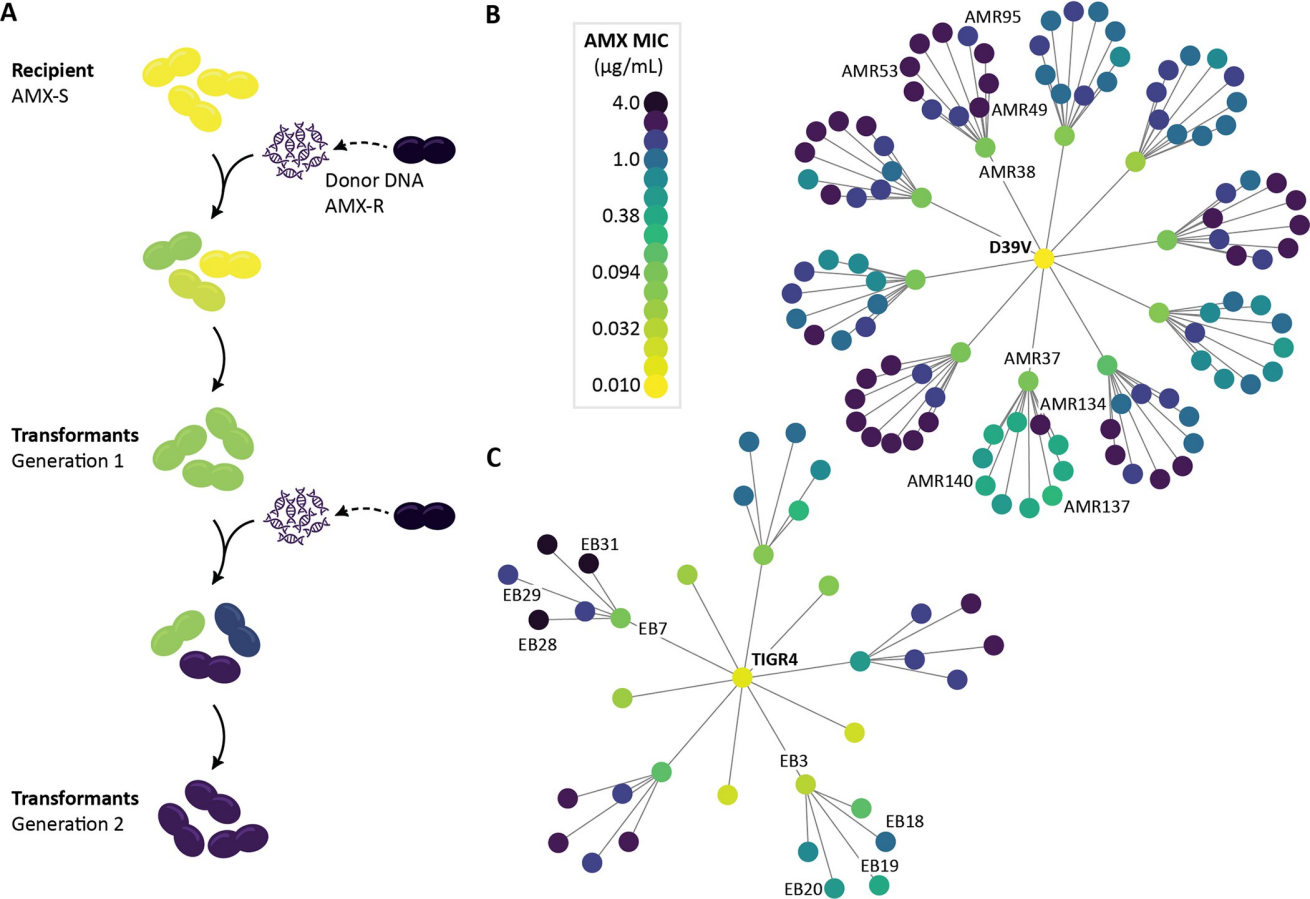
Transformation as an experimental tool to track the evolutionary path to AMX resistance. **(A)** Experimental design. Susceptible D39V and TIGR4 were transformed with genomic DNA from an AMX resistant clinical isolate then selected on a range of AMX concentrations. Ten colonies were picked at random from the highest concentration with growth (first round on 0.03 μg/mL and second round on 0.17–0.3 μg/mL, depending on MIC of the recipient strain). Isolated transformants with increased MICs were then subjected to a second round of transformation. **(B)** Pedigree chart of recombinant strains derived from D39V, and **(C)** from TIGR4. Color represents AMX MIC. Strains which were sequenced and used throughout the study are labelled.

In order to understand the sequence of recombination events leading to the development of AMX resistance, a susceptible *S*. *pneumoniae* strain D39V (minimum inhibitory concentration (MIC) 0.01 μg/mL) was transformed in two successive rounds with genomic DNA originating from AMX resistant clinical isolate 11A (MIC 4 μg/mL) followed by selection on a range of AMX concentrations above MIC (Fig 2A and Table 1). In the first round, ten colonies were picked at random from the highest AMX concentration with growth (0.03 μg/mL AMX). The MICs of these strains ranged from 0.047–0.125 μg/mL, 4–10 times that of wild-type D39V (Fig 2B and S1 Table).

**Table 1:** Key strains used in this study. The full collection of recombinant strains and their AMX MIC can be found in S1 Table.

Strain Name	Strain ID	Genotype and relevant characteristics	AMX MIC (μg/mL)	reference
D39V	VL1	Serotype 2 laboratory strain, AMX susceptible	0.001	[73]
TIGR4	VL2177	Serotype 4 laboratory strain, AMX susceptible	0.016	[124]
11A	SN75752 VL1313	Serotype 11A ST6521 clinical isolate, AMX resistant	4	German National Reference Center for Streptococci
AMR37	VL4481	D39V; recombinant strain, generation 1	0.094	This study
AMR38	VL4482	D39V; recombinant strain, generation 1	0.094	This study
AMR53	VL3196	D39V; recombinant strain, generation 2	2	This study
AMR95	VL4484	D39V; recombinant strain, generation 2	1.5	This study
EB31	VL4495	TIGR4; recombinant strain, generation 2	4	This study
D39V^2x37^	VL4496	D39V; *pbp2x37*	0.023	This study
D39V^2x38^	VL4302	D39V; *pbp2x38*	0.064	This study
D39V^2b37^	VL4497	D39V; *pbp2b37*	0.023	This study
D39V^2b38^	VL4498	D39V; *pbp2b38*	0.023	This study
D39V^2x37-2b37^	VL4499	D39V; *pbp2x37*; *pbp2b37*	0.094	This study
D39V^2x38-2b38^	VL3895	D39V; *pbp2x38*; *pbp2b38*	0.094	This study
D39V^M53^	VL3960	D39V; *murM53*	0.023	This study
D39V^1a53^	VL4159	D39V; *pbp1a53*	0.016	This study
D39V^1a95^	VL4160	D39V; *pbp1a95*	0.016	This study
D39V^2x38-2b38-M53^	VL3961	D39V; *pbp2x38*; *pbp2b38*; *murM53*	0.38	This study
D39V^2x38-2b38-M95^	VL3962	D39V; *pbp2x38*; *pbp2b38*; *murM95*	0.5	This study
D39V^2x38-2b38-1a53^	VL3896	D39V; *pbp2x38*; *pbp2b38*; *pbp1a53*	0.75	This study
D39V^2x38-2b38-M53-1a53^	VL3963	D39V; *pbp2x38*; *pbp2b38*; *murM53*; *pbp1a53*	2	This study
D39V^2x38-2b38-M95-1a95^	VL4500	D39V; *pbp2x38*; *pbp2b38*; *murM95*; *pbp1a95*	1.5	This study
AMR53^2b31^	VL4174	AMR53; *pbp2b31*	6	This study
AMR95^2b31^	VL4184	AMR95; *pbp2b31*	6	This study

The second round of transformation resulted in 100 strains with MICs from 0.25 to 2 μg/mL (Fig 2B), lower than the donor strain, and a third round of transformation did not increase the MIC further. Interestingly, five of the ten lineages had seven or more strains with MICs of 1 μg/mL or higher. In contrast, the AMR37 lineage resulted in MICs from 0.25–0.5 μg/mL, with one exception at 2 μg/mL. These lineage-dependent differences in AMX MIC range (*p* = 5 x 10^−4^, Fisher’s Exact Test) suggested that mutations acquired in the first round contributed to the recombination events which occurred in the second round, and thus to the MICs of the resulting strains.

Similar lineage-dependent MIC patterns were seen when TIGR4 was used as the recipient strain (*p* = 1.00x10^-3^, Fisher’s Exact Test) (Fig 2B). Here, the AMX MICs of the first-generation ranged from 0.023–0.5 μg/mL, with one strain showing a more than 30-fold increase from wild-type TIGR4. In the second-generation, three recombinant strains reached the same MIC as the donor strain (4 μg/mL), suggesting potential key differences in the recipient strain genomes, or deleterious epistatic effects of AMX resistance mutations which affected resistance development through recombination.

### Cell lysis upon AMX treatment correlated with PBP affinities

**Fig 3:**
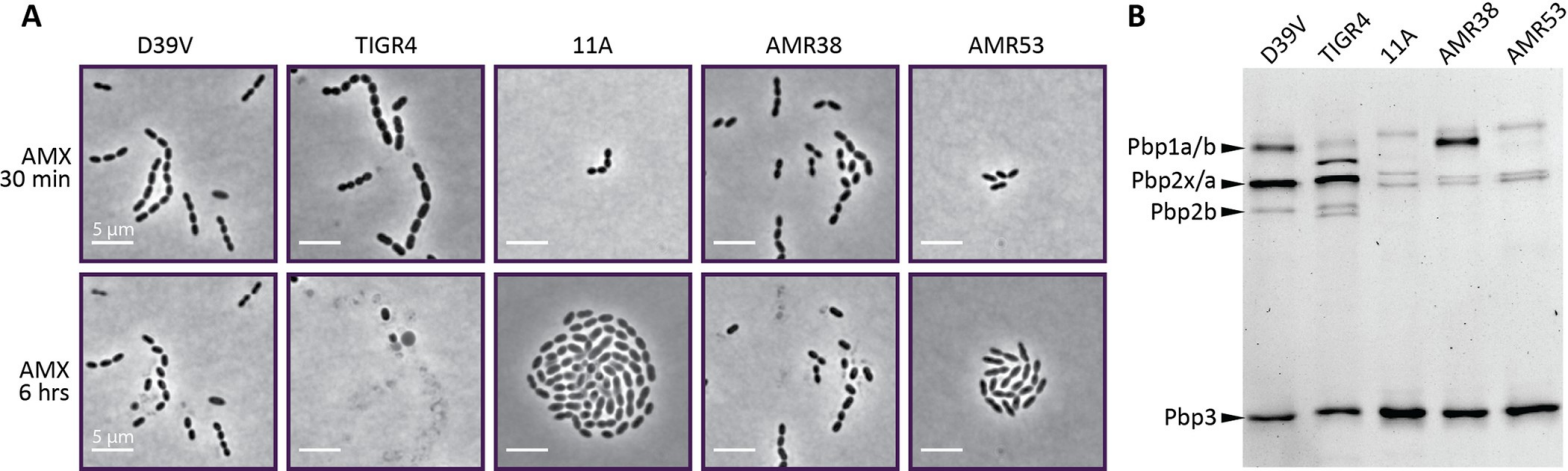
Phenotypic characterization of key strains used in this study. (**A**) Phase-contrast snapshots of recipient strains D39V and TIGR4, donor strain 11A, and recombinant strains AMR38 (generation 1) and AMR53 (generation 2) on C+Y agarose pads after 30 minutes and 6 hours of treatment with 1 μg/mL AMX. This concentration was above MIC for D39V, TIGR4, and AMR38, and lead to a complete stall in growth and partial lysis. Donor strain 11A grew well in this AMX concentration, although some heterogeneity in cell shape could be observed. Although lower than the AMX MIC for AMR53, this strain showed decreased growth and abnormally elongated cells, indicating cells were stressed. Scale bar is 5 μm. (B) Bocillin-FL binding patterns for the same five strains. Expected PBP band sizes for D39V are shown on the left. Image of Coomassie stained gel can be found in S1 Fig).

To test whether transformed strains phenocopy the donor with regards to AMX resistance, phase contrast microscopy with an AMX concentration above the recipient MICs (1 μg/mL) was performed. A complete stall in growth was observed for both recipient strains and first-generation recombinant AMR38, with mild to severe lysis after 6 hrs of treatment (Fig 3A). In contrast, donor strain 11A grew to form a microcolony in this AMX concentration, while second-generation AMR53 grew more slowly and with abnormally elongated cells (Fig 3A).

To test whether the increased resistance of AMR53 is related to alterations in the affinity of the PBPs towards AMX, we performed Bocillin-Fl labelling. Bocillin-Fl is a fluorescently labelled penicillin derivative that binds all six PBPs of *S*. *pneumoniae* [71], and the intensity of labelling can be used as a proxy for the affinity of the PBP for β-lactam antibiotics [72]. All six PBPs of D39V and TIGR4 had a high affinity for the fluorescently labelled penicillin (Fig 3B). Interestingly, TIGR4 showed some evidence of Pbp1a degradation products that bound Bocillin-Fl with high affinity even though it is 99.7% identical to D39V Pbp1a. In contrast, 11A showed an altered PBP pattern, where Pbp2b and Ppb1a showed complete loss of affinity, and Pbp2x was labelled more faintly and migrated more slowly (Fig 3B).

Both first- and second-generation recombinants demonstrated a loss of affinity of Pbp2b and Pbp2x for Bocillin-FL, with Pbp1a in AMR53 also showing reduced affinity, and potentially some degradation (Fig 3B). Together, these characterizations demonstrate that transformed recipients phenocopy the donor AMX resistant strain and have acquired PBPs with reduced affinity towards bocillin-FL.

### Recombination events clustered around resistance-associated loci

In order to examine the genetic differences in recombinant strains that may explain the differences in MIC, we selected eight recombinant strains from each experiment, and Illumina sequenced to ~600-fold mean coverage. For the D39V experiment, we chose AMR38 and three of its descent strains, as this lineage had the highest MICs. To contrast, we also chose AMR37 and three descendent strains, which had the lowest and most variable MICs in the second generation (Fig 2B). We used similar selection criteria for the TIGR4 experiment, taking strains from the high MIC EB7 lineage, and from the low MIC EB3 lineage (Fig 2C).

To be able to accurately map recipient to donor, we also performed long-read PacBio sequencing followed by short-read polishing on the 11A donor and TIGR4 recipient (an accurate genome map was already available for D39V [73]). Recombination was detected using SNPs, as described in [11]. On average 0.38% ± 0.22% of the donor genome was transferred during a single transformation event, and the total percentage transferred after two rounds was 0.80 ± 0.05%. Although higher numbers of recombination events were detected in the D39V dataset, the average event length was larger in the TIGR4 dataset (D39V 0.79 Kb and TIGR4 2.29 Kb, S3 and S4 Tables), resulting in similar total amounts of donor genome transfer after two rounds of transformation (D39V 0.79 ± 0.23%, TIGR4 0.80 ± 0.16%, S3 and S4 Tables). Of note, the longest recorded recombination fragment was 4.00 Kb for D39V and 12.52 Kb for TIGR4.

**Fig 4:**
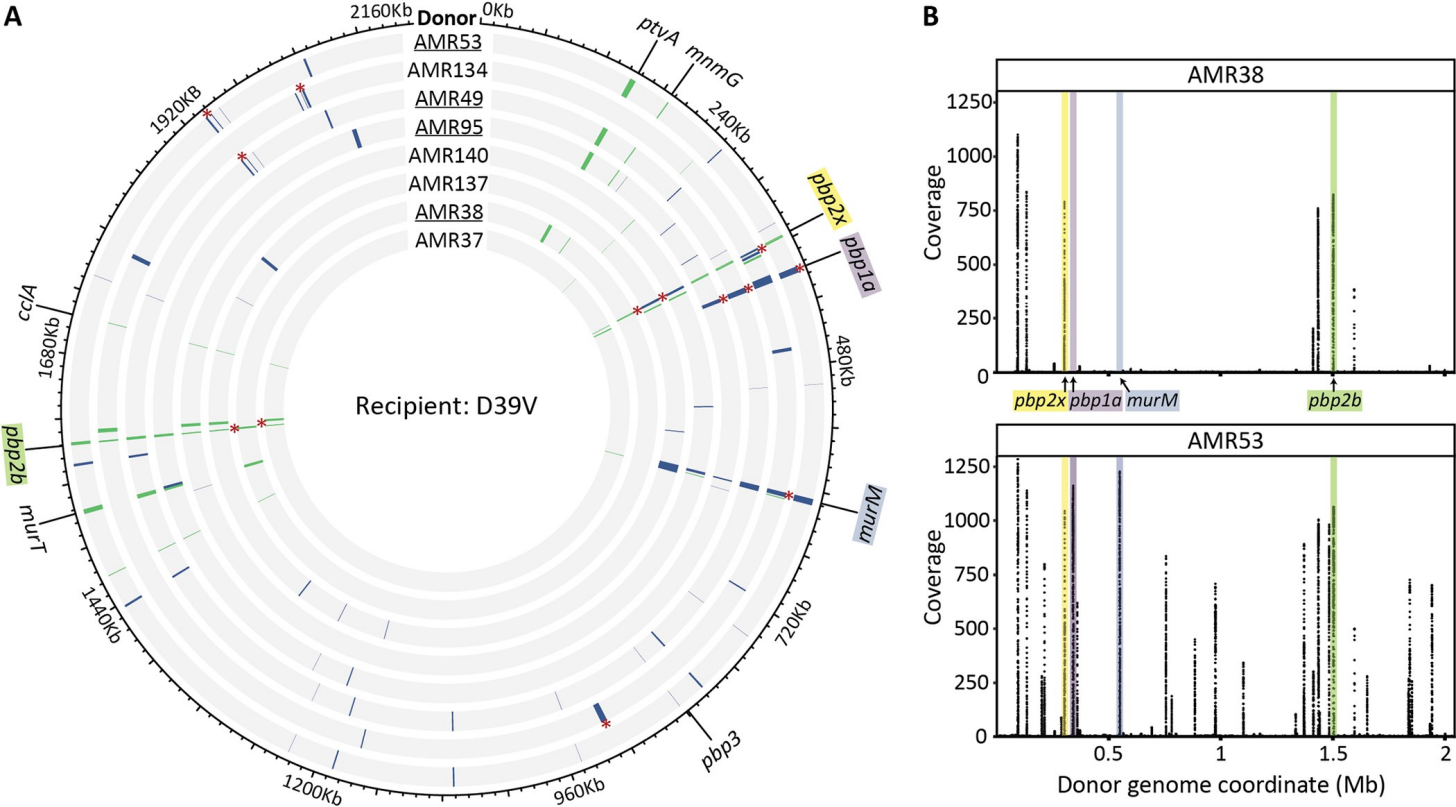
Widespread transformation and uptake of 11A donor DNA in recipient D39V upon AMX selection. **(A)** Circos plot with detected recombination events in D39V derived strains, arranged from lowest to highest MIC from the center. AMR38 and all derived strains are underlined, AMR37 and all derived strains are not. Grey indicates the sequences matches that of the recipient D39V, while recombination events are colored. Events acquired in the first round of transformation are shown in green, in the second round in blue, and lengths were increased to aid in visualization (see S3 Table for a list of all detected events). Recombination events determined to be non-contiguous are marked with (*). Non-contiguous events detected in the first-generation were removed from the data set on the second-generation strains, so are not marked. **(B)** Coverage of reads mapped competitively to the donor genome and plotted by coordinate beginning at the origin. Recombination events cluster at known β-lactam resistance determinants, *pbp2x*, *pbp2b*, *murM*, and *pbp1a*.

**Fig 5:**
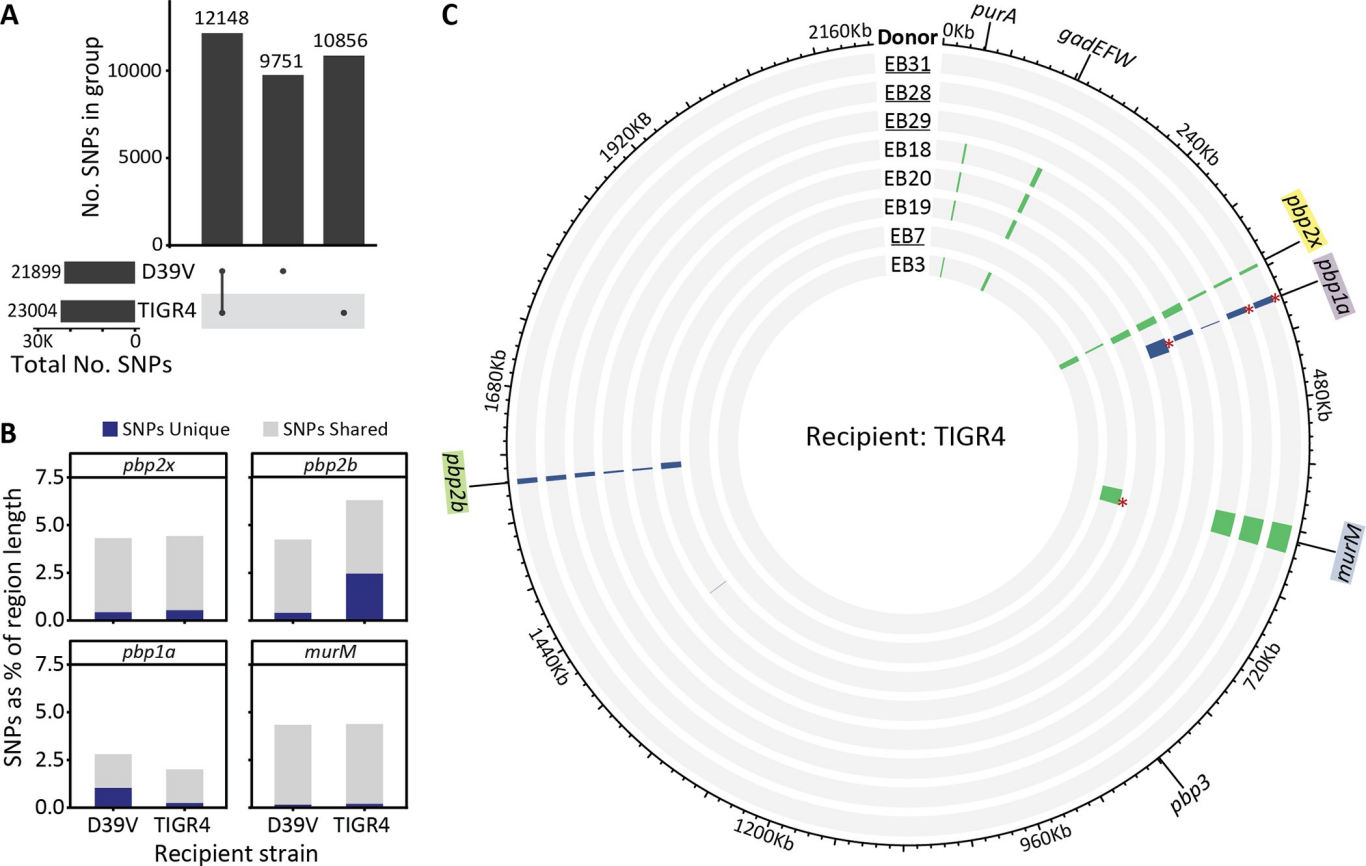
Uptake of four donor fragments is sufficient for AMX resistance in TIGR4. **(A)** Upset plot of shared and unique SNPs between each recipient and the donor. Bars on the left show the total number of SNPs for each recipient genome compared to the donor. In the central bar plot, from left to right, SNPs shared between D39V and TIGR4, SNPs found only in D39V, and SNPs found only in TIGR4. Although the two recipients differ from the donor strain by similar amounts of SNPs, only about half are unique. **(B)** SNP counts in *pbp/murM* genes, including 5 Kb up- and downstream. Number of SNPs unique to each recipient are shown in blue, and shared in light grey. **(C)** Circos plot showing detected recombination events in TIGR4 derived strains, arranged from lowest to highest MIC from inside to out. EB7 and all derived strains are underlined, EB3 and all derived strains are not. Grey indicates the sequences matches that of the recipient D39V, while recombination events are colored. Events acquired in the first round of transformation are shown in green, and in the second round in blue, and lengths were increased to aid in visualization (see S3 Table for a list of all detected events). Only six loci with recombination were identified in the entire data set, four of which are known β-lactam resistance determinants, *pbp2x*, *pbp2b*, *murM*, *pbp1a*.

Recombination events clustered around key loci for pneumococcal β-lactam resistance in both datasets; *pbp2x*, *pbp2b*, *pbp1a* and *murM* (Figs 4A and 5C). SNPs were also found at closely located loci, likely due to linkage. In the D39V strains, there was extensive recombination at distantly located sites. Examples include *ptvABC*, associated with vancomycin tolerance [74], and *murT*, which performs an amidation step essential for peptidoglycan crosslinking [75,76].

In contrast, of the six total recombination loci in the TIGR4 dataset, only three were located distally from the *pbp* and *murM* genes, and all were detected based on the presence of very few SNPs.

We hypothesised that differences in recombination patterns could have arisen from reduced similarity with the donor genome, however comparing total shared and unique SNPs did not suggest major differences in variability between the two recipient genomes (Fig 5A). When considering variation around key loci, we saw that the TIGR4 *pbp2b* region was more divergent from the donor than D39V. The quality of donor DNA used may have also played a role. Together, these results demonstrate that regardless of the recipient strain, four loci are consistently acquired in high AMX resistant recombinants, namely *pbp2x*, *pbp2b*, *pbp1a* and *murM* (Fig 5B).

Structural variation and base modification detection with PacBio sequencing revealed no major structural changes or differences in methylated motifs in the D39V recombinant strains. However, a local rearrangement was identified at the *hsdS-creX* locus in EB7, EB28, and EB29. In this region, spontaneous reshuffling of *hsdS* genes by CreX results in different methyltransferase specificity of the type I restriction modification system SpnD39III [73,77,78]. TIGR4 was found to be predominantly in the B-configuration, EB7 and EB29 in the D-configuration, and EB28 in the C-configuration, while all D39V-derived strains and 11A were mostly in the F-configuration. Differences in methylated motifs have transcriptomic consequences [77], however MIC differences among these recombinants can largely be explained by SNPs in key loci for β-lactam resistance.

### Non-contiguous recombination events likely result from a single donor molecule

Recombination is often thought of as a donor molecule forming a single D-loop at a homologous site on the recipient chromosome, which is then expanded in both directions until the requirement for homology is no longer met, at which point the interaction ends. However, we detected recombination events from one round of transformation located very close together on the chromosome, sometimes within the same gene but with separating recipient SNPs, a motif reminiscent of clinically resistant mosaic *pbp* alleles. This has also been noted previously in both *in vitro* pneumococcal and *H*. *influenzae* recombination studies and was hypothesized to result from a single donor molecule [8,70,79]. To determine if events were significantly close together, forming a single non-contiguous recombination event, a bootstrapping approach was applied to the distances between novel recombination events within the same strain [8]. In this approach, the shortest distances between all recombination events for all strains were pooled to form the test population. Bootstrap tests were performed 1000 times, where the test population was sampled with replacement, and the resulting distribution compared to the shortest recombination distance for each strain (*d_test_*). If *d_test_* was below the 0.05 quantile value in at least 95% of bootstrap tests, then the events were considered to be linked, and the next closest pairing was tested. We found 37 events across all strains that could be amalgamated into single events under the assumptions of this approach (S2 Fig and S4 Table).

In addition, using PCR amplified fragments of the *pbp2x-mraY* locus from 11A, we were able to test, for the first time, whether non-contiguous recombination events occurred more frequently when the template was provided as a single molecule, or as two overlapping fragments. By ligating additional regions of non-homologous DNA, we also tested the effect of molecule length on recombination.

**Fig 6:**
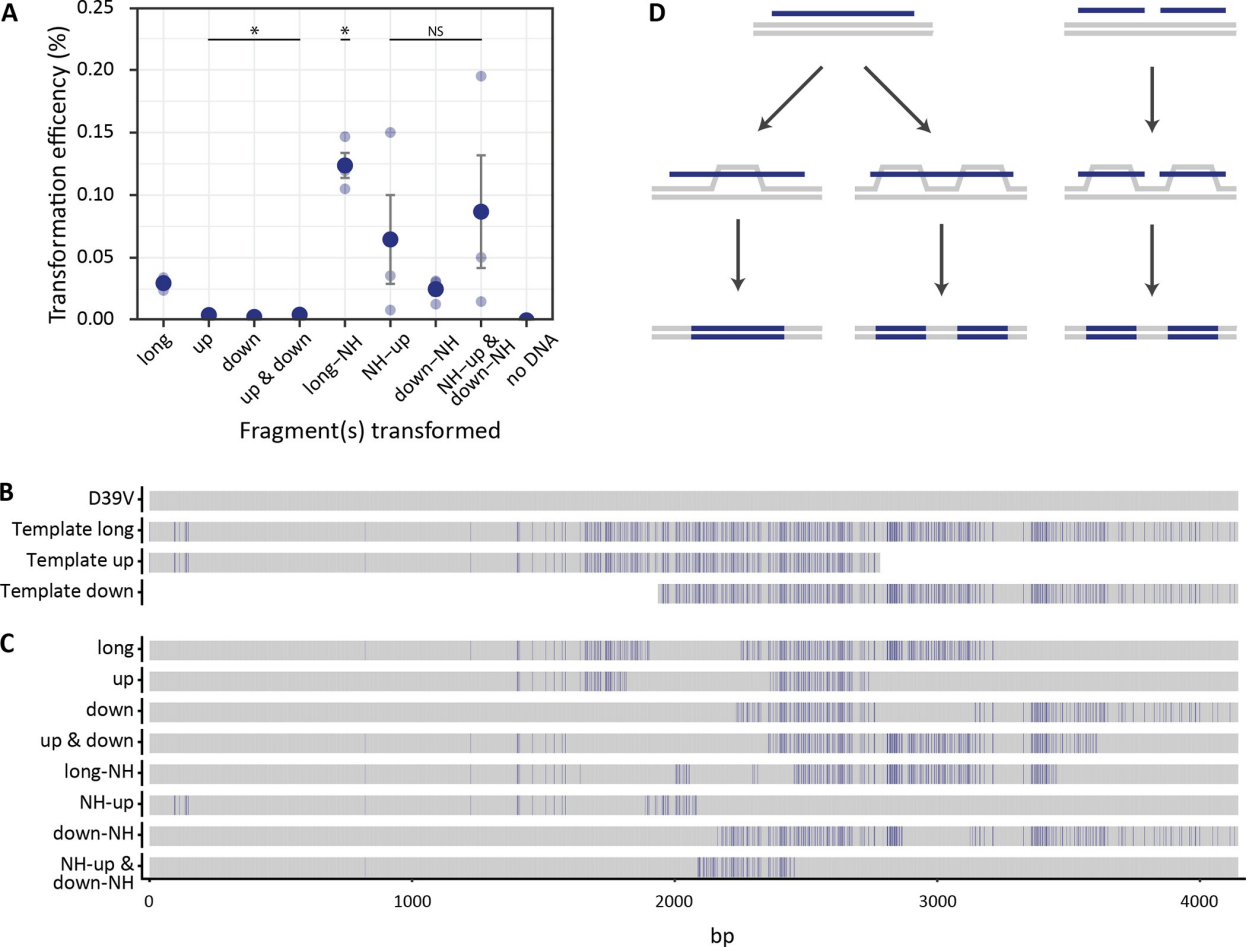
Fragmented transformation of donor DNA predominantly originates from non-contiguous recombination events. Three fragments were PCR amplified from the 11A strain *pbp2x* locus, one long 4.2 Kb fragment, and two overlapping short fragments (up 2837 bp, and down 2260 bp). In addition, the same fragments were ligated to stretches of non-homologous DNA, taking all total lengths to 6 Kb (long-NH, NH-up, and down-NH). **(A)** Efficiencies of transformation of fragments into D39V with selection on AMX (0.015 μg/mL). Statistically significant differences in efficiency compared to the long fragment was determined by *t* test with BH correction for multiple testing. NS not significant, * indicates *p* < 0.05. **(B)** Alignment of the homologous region of template fragments used in the experiment. Donor SNPs are shown in blue, while bases matching the recipient (D39V) are shown in grey. **(C)** Alignment of 4.2 Kb locus from colonies transformed with different fragments. Bases shared with donor but not with recipient are shown in blue. **(D)** Schematic showing hypothesized molecular mechanisms for non-contiguous and contiguous recombination. As shown in panel B, both events can take place, but based on transformation efficiencies, the process on the left with long donor DNA is more efficient.

Transformation efficiency increased with length of both the overall molecule, and the homologous region (Fig 6A). This is in line with previous studies showing the importance of molecule length and homology in pneumococcal transformation [80,81]. We hypothesize that increased length may increase the likelihood of spontaneous contact with the ComGC pilus, while also decreasing the likelihood of homologous region fragmentation by EndA, prior to entry [82]. The efficiencies of the short overlapping fragments were lower than that of the long fragments, although increased slightly when transformed in combination.

Recombination occurred in all colonies sequenced. When two overlapping fragments were provided, uptake was often unequal, with either the upstream or downstream fragment incorporated more often. Recombination of both fragments was observed in 1/20 cases and was not observed when fragments were lengthened by non-homologous DNA (S3–S5 Figs and S7 Table).

Non-contiguous recombination was detected in all conditions, although the frequency of occurrence increased with length of homology (Figs 6C and S3, S4, S5 and S7 Table). It was observed both when the template was given as a single long fragment, and as two overlapping fragments.

Overall, the results suggested that the formation of multiple D-loops from either a single template molecule, or from multiple molecules, could lead to non-contiguous recombination events (Fig 6D). However, given that longer homology and total molecule length increased the efficiency of transformation partly by reducing strand degradation [81], there may have been more opportunity for complex recombination patterns to occur when longer fragments were taken up.

### *AMX resistant* pbp *and* murM *alleles are sufficient to explain AMX MICs*

Substitutions in all four proteins (Pbp1a, Pbp2b, Pbp2x and MurM) were required to reach an MIC close or equal to that of the donor strain (S6 Fig and S5 Table), but the order of uptake varied both stochastically, and depending on the recipient genome. The correlation between allele uptake and MIC increase was consistent in the D39V background. First-generation strains had mutations in *pbp2b* and *pbp2x*, the two essential PBPs for cell growth [64]. In the second-generation, all strains acquired mutations in *murM*, and those with MICs higher than 1 μg/mL also had mutations in *pbp1a*. In TIGR4, *pbp2x* alleles were also acquired in the first-generation, however *pbp2b* mutations only appeared after the second round of transformation. Mutations in *murM* were recombined in EB7 in the first round of transformation, then were inherited vertically in that lineage, but were not acquired by EB3 or any descendant strains (Fig 5C). Although *murM* mutations were not present in all strains with *pbp1a* modifications, strains with recombination events at all four loci had higher MICs (Fig 2C). This indicated that *murM* mutations may be less important for successful integration and selection for *pbp1a* mutations but are key for expression of the AMX resistant phenotype of the donor [60–63].

**Fig 7:**
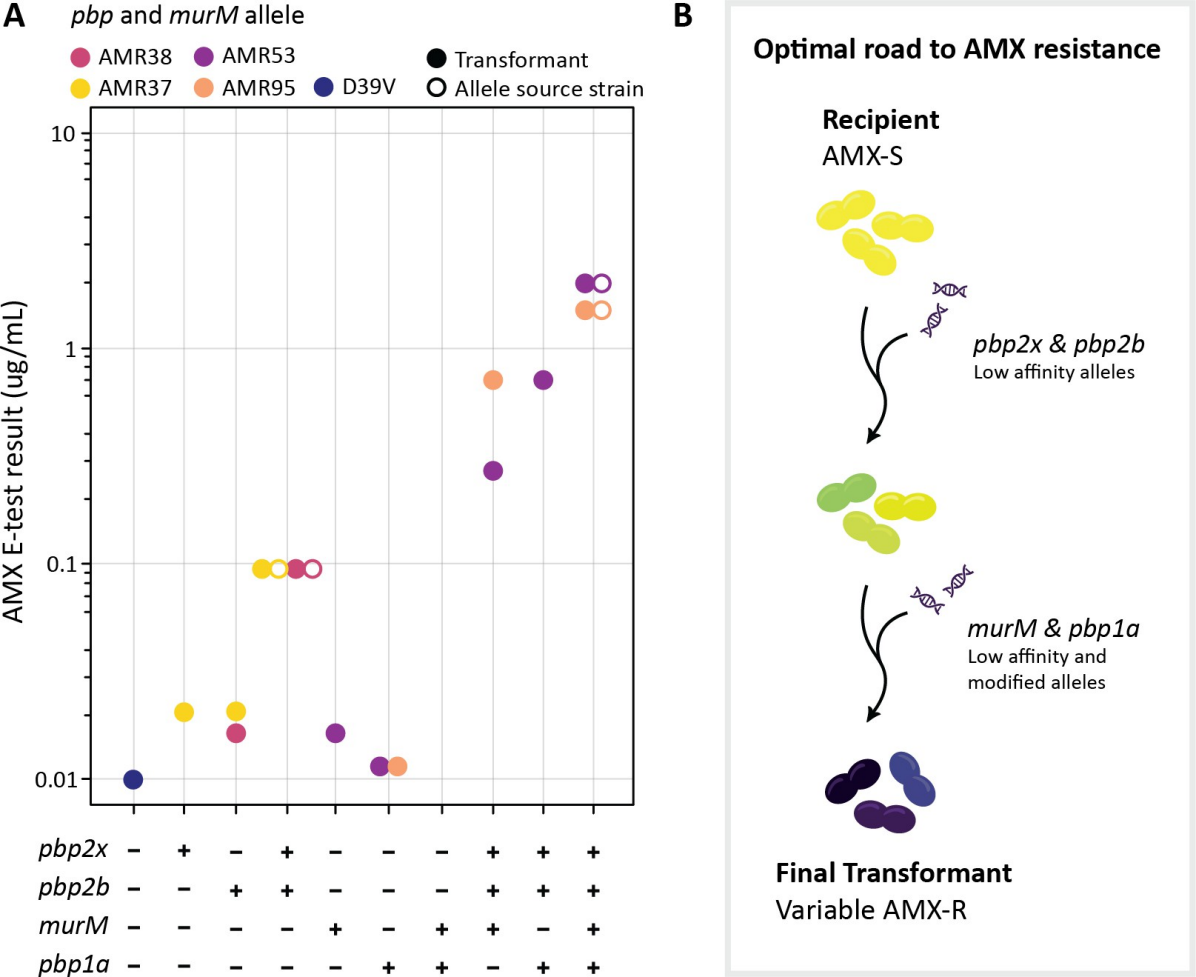
Uptake of mosaic *pbp1a*, *pbp2b*, *pbp2x* and *murM* is required and sufficient for clinically relevant amoxicillin resistance in *S*. *pneumoniae*. (A) AMX MICs determined by E-test for D39V transformant strains (solid circles) where *pbp* and *murM* alleles from different source strains (empty circles) were transformed in combination. Color shows allele source. (B) Schematic overview of the optimal road to AMX resistance development through horizontal gene transfer. Low affinity *pbp2x* was taken up in the first round of transformation in both datasets, whereas donor *murM* and *pbp2b* alleles were acquired in the first or second round, depending on recipient and lineage. Low affinity *pbp1a* was recombined in the second round, and only found in strains with AMX MICs above 0.75 μg/mL. In principle uptake of low affinity and modified alleles could be achieved in a single (unlikely) or double step.

An experiment was designed to test whether the order of allele uptake in D39V recombinant strains was crucial for AMX resistance development, and if PBP and MurM substitutions alone were sufficient to explain AMX MICs. Alleles of *pbp2x* and *pbp2b* from AMR37 (*pbp2x37*, *pbp2b37*) and AMR38 (*pbp2x38*, *pbp2b38*) were transformed into D39V and selected on AMX. Strains carrying either a recombinant *pbp2x* or *pbp2b* allele had E-test MICs up to 0.064 μg/mL. However, when both *pbp* alleles were integrated (D39V^2x38-2b38^), the E-test result equalled that of the recombinant allele donor (Fig 7), suggesting that mutations outside these two loci were not involved in the observed reduction in AMX susceptibility in the first-generation strains.

**Fig 8:**
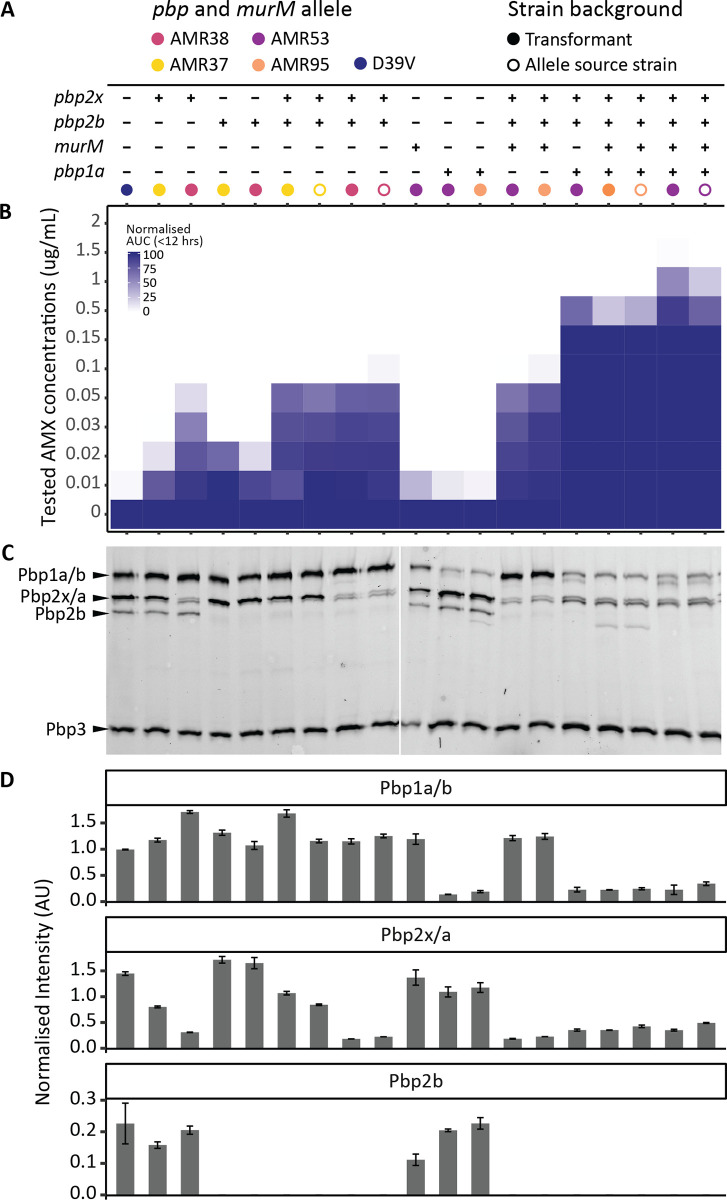
Recombinant PBP proteins visualized by PBP-bocillin binding affinities. Transformed AMX resistant strains produce mosaic PBPs with reduced affinity for Bocillin-FL. Each column shows the phenotypic characterization of one *pbp* and *murM* allele swap strain. **(A)** Table showing the presence (+) and absence (-) of alleles from different source strains (colour). Allele source strains are also included and are indicated by the unfilled circles. **(B)** Survival in different AMX concentrations quantified by determining the area under the growth curve (AUC) after 12 hours growth in AMX normalized by AUC in the absence of AMX. **(C)** Bocillin-FL binding affinity gel. **(D)** Intensity of bands corresponding to Pbp1a/b, Pbp2x/a, and Pbp2b, normalized by the intensity of the Pbp3 band of the same lane, as this allele remained unmodified in all strains.

To assess whether mutated *pbp2x* and *pbp2b* were critical for *murM* mutation uptake, D39V was transformed with *murM* from both AMR53 (*murM53*) and AMR95 (*murM95*) and MICs determined. Surprisingly, we were able to select for colonies with integration of the AMR53 *murM* allele (D39V^M53^) resulting in an E-test result of 0.023 μg/mL, despite this protein not being a direct target of AMX (Figs 7 and 8A and 8B). The genome was sequenced, and no spontaneous point mutations were identified in previously described resistance determinants outside *murM* (S8 Table). When transformed into the D39V^2x38-2b38^, the E-test MICs reached 0.5 μg/mL and 0.38 μg/mL for AMR95 and AMR53 *murM* alleles (D39V^2x38-2b38-M95^, D39V^2x38-2b38-M53^), respectively. AMX susceptibility decreased substantially when all three genes were mutated, however failed to reach the final MIC of the recombinant allele donor, indicating the necessity of another resistance determinant (Fig 7).

*pbp1a* alleles from AMR53 and AMR95 (*pbp1a53*, *pbp1a95*) could not be successfully selected on AMX when transformed into D39V, either alone or in conjunction with a *murM* allele from the same donor. Indeed, Pbp1a is not a key target of AMX, and this result was not unexpected. We instead transformed a construct where a kanamycin resistance cassette was cloned behind *pbp1a*, with 1 Kb of homology up- and downstream. Growth in liquid culture of the resulting strains at AMX 0.01 μg/mL was similar to D39V (Fig 8A and 8B). Interestingly, successful integration occurred for both *pbp1a53* and *pbp1a95* when transformed into D39V^2x38-2b38^ (D39V^2x38-2b38-1a53^, D39V^2x38-2b38-1a95^), suggesting that a mutated *murM* allele was not required for mutations in *pbp1a* to cause a selectable increase in AMX MIC. However, substitutions in Pbp2x, Pbp2b, and Pbp1a alone were insufficient to replicate the E-test results of the allele source strains (Fig 7).

We therefore transformed *murM* and *pbp1a* alleles from AMR53 and AMR95 into D39V^2x38-2b38^ simultaneously, and found that selection was possible on higher concentrations of AMX (1 μg/mL) than for either gene alone (0.2 μg/mL). The resulting strains (D39V^2x38-2b38-M53-1a53^, D39V^2x38-2b38-M95-1a95^) had AMX resistance phenotypes matching those of AMR53 and AMR95, with E-test results of 2 μg/mL and 1.5 μg/mL respectively (Fig 7).

The strains containing all four genes from AMR53 and AMR95 were whole genome sequenced to check for spontaneous point mutations outside these loci. One non-synonymous mutation in *glnA* (glutamine synthase type I) was found in both strains. Downregulation of *glnA* has been linked to the pneumococcal penicillin stress response [83], but no differences in AMX survival were observed in these strains compared to the allele donors lacking the SNP.

To quantify the affinities of resistant PBP alleles to β-lactam antibiotics, Bocillin-FL binding patterns were determined for allele swap strains and the source strains (Fig 8C and 8D). This confirmed that the recombinant PBPs had reduced penicillin binding affinities. For Pbp2b, both the AMR37 and AMR38 alleles resulted in almost complete loss of binding, and a band could not be quantified (Fig 8D). A large reduction in affinity was observed for Pbp2x38, larger than for Pbp2x37, which correlated to a small difference in growth at 0.1 μg/mL AMX between the two source strains (Fig 8A and 8B). This could be explained by 13 amino acid substitutions including S389L and T338A which were not present in the AMR37 allele but have previously been linked to reduced susceptibility to β-lactams [55,72].

Perhaps most interesting were the two low affinity Pbp1a alleles tested. In all strains containing *pbp1a95* (AMR95 derived), reduced band intensity was observed in the Pbp1a/Pbp1b size range, however, a faster migrating species was also detected. The *pbp1a95* allele contained a spontaneous point mutation (not present in the 11A donor) causing a premature stop codon. This resulted in the loss of 65 amino acids (of a total of 720) and put the predicted size at 73.1 kDa instead of 79.6 kDa. Reduced intensity at the expected Pbp1a/Pbp1b size range was also observed for the AMR53 allele (Pbp1a53) however, an additional Bocillin-FL-tagged species which migrated slightly faster than expected was also observed. The predicted protein length of Pbp1a53 is identical to D39V and the donor, with no premature stop codons identified. The additional band may indicate some degradation of this protein species, although the TP domain must still be available for Bocillin-FL binding. Together, these experiments demonstrate that transfer of the *pbp2x*, *pbp2b*, *pbp1a* and *murM* alleles of 11A into strain D39V is sufficient to explain the observed AMX resistance in transformants obtained using chromosomal DNA.

### High AMX resistance in the D39V comes at a significant fitness loss

**Fig 9:**
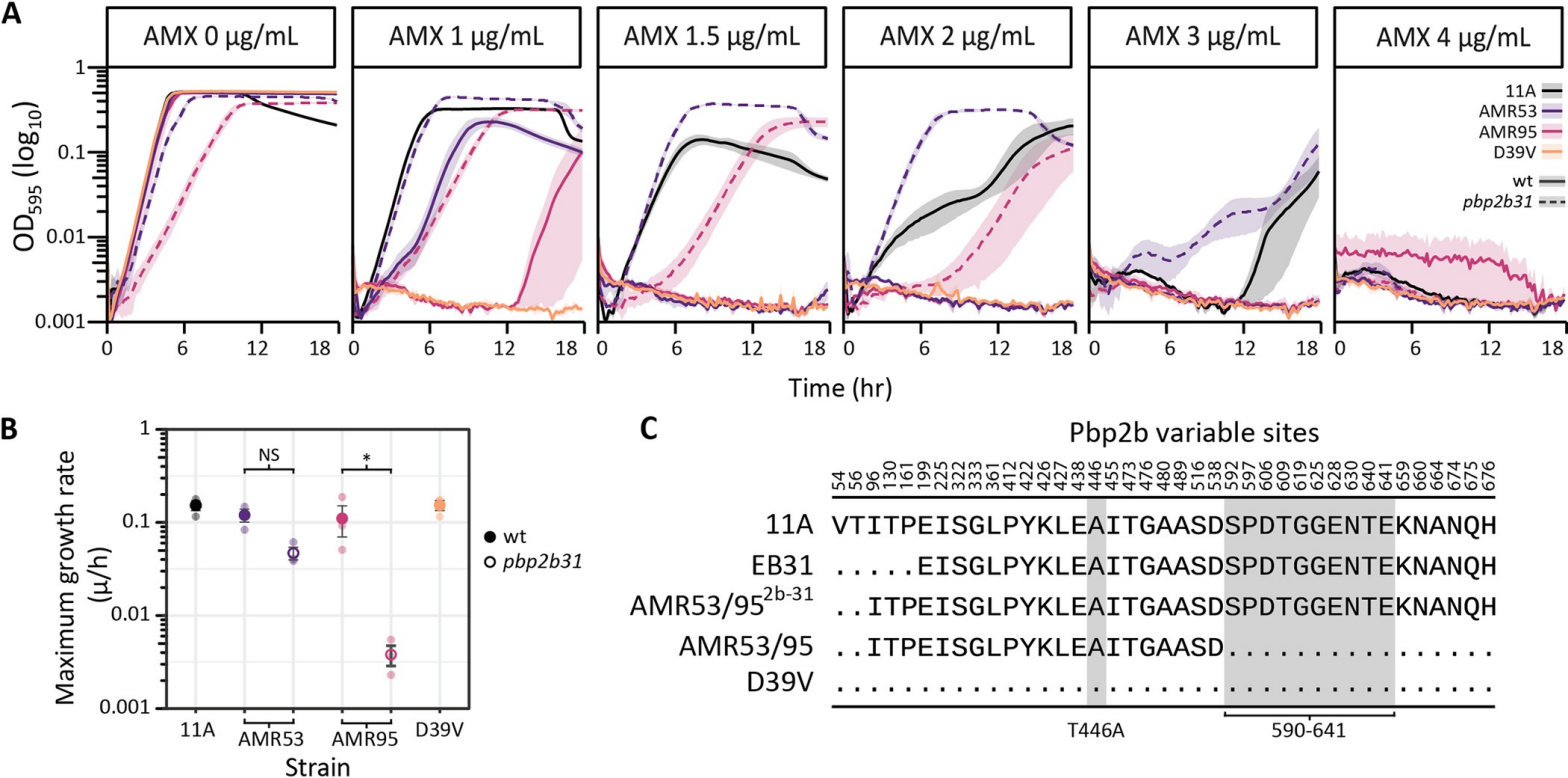
Ten substitutions in Pbp2b 590–641 region essential for high AMX resistance. **(A)** Growth of AMR53^2b31^ and AMR95^2b31^ (dotted lines) in different AMX concentrations. 11A, D39V, AMR53, and AMR95 (solid lines) are shown as controls. **(B)** Dot plot of maximum specific growth rates (μ/h) calculated from the AMX 0 μg/mL condition in triplicate (transparent circles). The mean for each strain is shown by an opaque circle, solid for wild-type (wt) strains and empty for AMR53^2b31^ and AMR95^2b31^. Color denotes strain background. Significance was determined by one-way ANOVA with a Tukey correction for multiple testing. **(C)** Amino acid sites in pbp2b which differ from D39V for strains 11A, EB31, AMR53 (identical to AMR95), and AMR53^2b31^ (the result of transforming AMR53 or AMR95 with the EB31 pbp2b allele). Dots indicate the amino acid matches D39V, and key substitutions, T446A and a block of 10 between 590–641 [54–56], are highlighted in grey.

Of the 16 recombinant strains sequenced, only EB31 and EB28 from the TIGR4 collection had the same AMX MIC as the donor strain, and a third round of transformation with gDNA into AMR53 failed to increase the MIC. The only region of mutations that EB28 and EB31 had in common that was not present in the highest MIC AMR strains, was a block of 10 mutations in the 590–641 region of Pbp2b (Fig 9C and S5 Table). To test whether these substitutions were sufficient to explain the MIC difference, the *pbp2b* allele from EB31 (*pbp2b31*) was transformed into AMR53 and AMR95. The AMX E-test scores for the resulting strains were both 4–6 μg/mL. However, growth curves revealed a significant fitness loss, with a mean maximum specific growth rate of 0.11 μ/h for AMR95 decreasing almost 100-fold to 0.0036 μ/h for AMR95^2b31^, which was exacerbated by the presence of AMX (Fig 9B). In contrast, AMR53^2b31^ grew almost 3-fold slower than AMR53 (AMR53 0.12 μ/h, AMR53^2b31^ 0.046 μ/h, Fig 9B), but showed a lytic phenotype in stationary phase. Interestingly, this strain outgrew the 11A donor in sublethal AMX concentrations (Fig 9A). The difference in growth rate between AMR95^2b31^ and AMR53^2b31^ was likely explained by the truncated Pbp1a95 protein. Significant modifications to the active sites of essential proteins often need to be compensated for, which may be impacted by the sub-optimal performance of other proteins in the system. Although the TP and TG domains of Pbp1a95 were intact, it is possible that the loss of 65 amino acids from the C-terminal resulted in an overall reduction in function, unrelated to its AMX binding affinity. This correlates with previous studies showing that compensation by substitutions in Pbp1a are required to mitigate fitness loss resulting from modifications to the Pbp2b active site [59]. Together, these experiments show that D39V is capable of reaching high AMX resistance, but that the absence of compensation by Pbp1a substitutions may result in significant fitness costs.

## Discussion

Natural competence and homologous recombination in *S*. *pneumoniae* allow for enormous genomic plasticity which contributes to the rapid development of vaccine-escape and antibiotic resistant isolates. In one round of transformation, more than 29 recombination events can occur in a single cell [11], while events up to 50 Kb in length have been identified in clinical isolates [5], allowing for significant remodelling at multiple loci simultaneously. Indeed, here we show a two-step sequential transformation experiment with up to 20 individual recombination events around the genome and lengths up to 13 Kb, resulting in an average donor genome transfer per step of 0.38%. This allows for huge genomic diversity, which, when placed under selective pressure, can lead to the rapid spread of antibiotic resistance-associated genotypes [84].

β-lactam resistance in the pneumococcus relies on DNA movement between closely related strains and species within the same niche, allowing tens of substitutions to accumulate in the target proteins [48]. It has previously been shown through *in vitro* transformation experiments into a laboratory strain that the main determinants of β-lactam resistance are modified alleles of *pbp2x*, *pbp2b*, *pbp1a*, and *murM* [61,85,86]. For the front-line antibiotic AMX, resistance has also been previously associated with mutations in these alleles although the relative importance of the changes in each locus is not well understood [54–57,60,61]. We confirmed the requirement for mutations in all four genes and showed that subtle differences between alleles could cause noticeable changes in both AMX-dependent fitness and the penicillin-binding affinity of PBPs.

In addition, a specific order of mutation uptake for penicillin resistance has been demonstrated in both *Staphylococcus aureus* and the pneumococcus [87–89], and for AMX this order was found to be *pbp2x*, *pbp2b*, then *pb1a*, with the role of *murM* unable to be confirmed in that experimental system [56]. Here, we confirm that substitutions in Pbp2x or Pbp2b are a critical first step for AMX resistance, perhaps reflecting the high affinity of Pbp2x for the antibiotic [72](Fig 7C), and that the T338A substitution is required for resistance to fully develop. We also find that *murM* mutations appear to be an intermediary step, and that a modified *pbp1a* allele is important for high-level AMX resistance. It is however important to note that these experiments were performed using high concentrations of naked DNA as a model, while in the nasopharynx genetic exchange likely occurs via fratricide or from small quantities of free environmental DNA [90,91]. It may be that lower donor DNA concentrations cause slower resistance allele acquisition, and result in lower fitness costs. In addition, previous studies have strongly implied the existence of multiple mechanisms of AMX resistance in *S*. *pneumoniae*, associated with different *pbp2b* and *murM* alleles [52,54,56], and that by using a single donor strain we were limited to the study of only one. However, we confirmed that a previously described block of ten substitutions in the 590–641 region of the Pbp2b TP domain [54–56] was essential to reconstruct the AMX MIC of the serotype 11A ST6521 donor strain in D39V and TIGR4. Perhaps most interestingly, we also show that the loss of 65 amino acids from the C-terminus of Pbp1a results in a significant fitness cost in the presence of these additional Pbp2b substitutions, highlighting the importance of interactions and fitness loss compensation between essential cell-wall synthesis proteins [59].

In addition to these definite experiments demonstrating the evolutionary route to clinical AMX resistance, we show that non-contiguous recombination could result from either a single, or multiple, donor DNA molecules. It is easy to imagine a long ssDNA-RecA presynaptic filament interacting with the chromosome at multiple points in the 3-dimensional cellular space [33,34](Fig 1 model), which then initiates the formation of several D-loops simultaneously as homologous DNA-DNA interactions stabilize [36,38,39]. On the other hand, SsbB coated ssDNA sequestered in the cytoplasm could provide a pool from which multiple donor molecules homologous for the same chromosomal region could be sourced [23]. However, we also observed that both transformation efficiency and non-contiguous event occurrence increased with length of homology and total length of the donor molecule, suggesting that single donor molecule events may simply be more likely. This concurs with the previous observation of transforming DNA concentration-independent differences in recombination event density, which would not have been expected under a multiple donor molecule hypothesis [8]. Recombination structures such as these have also been observed in *H*. *influenzae*, with the authors noting that localized clustering of long tracts of donor sequence appeared too close to have occurred by chance [70]. They hypothesized incoming DNA degradation via cytosolic or translocation endonucleases resulted in these clusters. In addition, mismatch repair via the Hex system could in principle be responsible for the reversion of small numbers of point mutations between non-contiguous recombination segments, although the system was likely saturated in our model [46]. To untangle the different possible mechanisms of non-contiguous recombination from other effects on transformation efficiency, testing mixtures of different donor fragment lengths would be necessary.

Altering the active sites of essential proteins in cell division is risky and requires a delicate balance between antibiotic avoidance and fitness loss. Accumulation of sufficient mutations in the appropriate order is aided both by the simultaneous recombination of multiple donor fragments distally around the genome, and by the occurrence of complex non-contiguous events. The experimental transformation experiments combined with whole genome sequencing as performed here provide valuable insights into viable and non-viable evolutionary paths toward AMX resistance development in the pneumococcus. The strong co-dependence among different proteins and their alleles creates significant challenges for complete understanding of resistance mechanisms, and emphasizes the importance of combining data from phenotypic, molecular, genomic, and genome-wide association studies.

## Methods

### Bacterial strains, growth conditions, and antibiotics

*S*. *pneumoniae* strains are shown in Tables 1 and S1. Bacteria were cultured in liquid C+Y medium with no shaking at 37°C. C+Y media was adapted from Adams and Roe [92].

For transformation, *S*. *pneumoniae* was grown in C+Y (pH 6.8) at 37°C to OD_595_ 0.1. Competence was induced by addition of 100 ng/ml synthetic CSP-1 (D39V) or CSP-2 (TIGR4) and 12 min incubation at 37°C. DNA uptake occurred during 20 min at 30°C, followed by dilution and recovery for 1.5 hr. Transformants were selected by plating inside Columbia agar supplemented with 3% defibrinated sheep blood (CBA, Thermo Scientific) containing the appropriate antibiotic and incubated at 37°C in 5% CO_2_ overnight. Successful transformants were confirmed by PCR and Sanger sequencing (Microsynth).

Strains were stocked at OD_595_ 0.3 in C + Y with 15% glycerol at -80°C.

AMX (Sigma Aldrich) powder was dissolved initially in 100% DMSO. This solution was diluted in molecular grade water with 4% DMSO to two concentrations, 100 μg/mL and 1 mg/mL, and stored at -80°C.

### Minimum inhibitory concentrations (MICs)

Bacteria were grown in C+Y broth to OD_595_ 0.1, then AMX MIC was determined using E-tests (Biomeriaux, Lyon) on Mueller Hinton agar containing 5% defibrinated sheep’s blood. MICs were read after 18 h incubation at 37°C and 5% CO_2_.

Strains with MICs below the limit of the E-tests (0.016 μg/mL) were tested in Mueller Hinton cation-adjusted broth with 5% defibrinated sheep’s blood using the broth micro-dilution method [93]. Briefly, bacteria were streaked onto MH2-blood plates and grown overnight. Colonies were picked from plates and ressupended in PBS (pH 7.4) to McFarlane standard 0.5. This inoculum was diluted 100-fold into 96-well plates containing MH2-blood broth and AMX (in 2-fold concentration steps) then plated on agar plates as a control for contamination and cell density. Plates were incubated for 18 hours at 37°C and 5% CO_2_. MIC was defined as the lowest concentration of antibiotic where visible α-haemolysis occupied approximately less than 10% of the surface area of the well. Due to a high level of subjectivity, MIC determination was repeated twice on different days, and MIC calls were confirmed by a second person in each case.

### Growth curves and analysis

Strains were precultured in C+Y broth to OD 0.1, then diluted 100-fold into 96-well microtiter plates, containing fresh C+Y and AMX. The OD_595_ was measured every 10 min for 12 hr in a plate reader (Infinite F200, Tecan) with incubation at 37°C. All strains were tested in triplicate

The area under the curve (AUC) of each replicate was divided by that of the no antibiotic control for each strain, to normalize for strain-dependent differences in growth. The mean and standard error of the mean (SEM) of the normalized AUC was determined for three biological replicates.

Statistical significance of maximum specific growth rate (μ/h) was determined using one-way ANOVA with a Tukey correction for multiple testing.

### Bocillin-FL binding assay

Bocillin-FL is a fluorescently labelled penicillin molecule which can be used to visualise and quantify PBP-penicillin binding affinity *in vitro*. Strains were grown to OD 0.2 in 4 mL C+Y, washed and resuspended in phosphate buffered saline (PBS), then incubated for 30 min at 37C in 5 μg/mL bocillin-FL (Invitrogen). Cells were washed in ice cold PBS, then lysed by sonication (10 x 1s pulses at 30% power). The cell lysate was spun down and supernatant removed to collect the membrane fraction, which was homogenized by sonication (1s pulse at 10%). Total protein concentration was determined by nanodrop, then samples were diluted in NuPAGE LDS sample buffer (Life Technologies) with 10 mM DTT and incubated at 95°C for 5 min. Samples were loaded into two 15-well Novex WedgeWell 10% Tris-Glycine 1.0 mm Mini Protein Gels (Invitrogen), to a total protein amount of approximately 1.8 mg in a maximum volume of 30 μL. Where necessary, gels were imaged simultaneously on an Amersham Typhoon (GE Healthcare) with Cy2 DIGE filter setup. Bands were quantified using ImageQuant (GE Healthcare). As all strains tested had identical Pbp3 proteins, the intensities of the Pbp1a/b, Pbp2x/a, and Pbp2b bands were divided by the PBP3 band intensity of the corresponding sample for normalisation. Imaging and quantification were performed in duplicate. Coomassie Blue staining was also performed, to confirm equal sample loading.

### Phase-contrast time-lapse microscopy

For time-lapse microscopy, cells were pre-cultured in liquid C + Y medium at 37°C and spotted on a low-melting 1.2% C + Y agarose patch, with or without AMX at 1 μg/mL. Phase-contrast pictures were taken every 10 min using a Leica DMi8 with a 100x phase contrast objective.

### Isolation of genomic DNA

10 mL of OD_595_ 0.2 culture was pelleted by centrifugation, then resuspended in Nuclei Lysis solution (Promega) supplemented with 0.05% SDS, 0.025% deoxycholate (DOC), and 200 μg/mL RNase A. This was incubated at 37°C for 20 minutes, 80°C for 5 minutes, then 37°C for 10 minutes. Protein Precipitation Solution (Promega) was added to the lysate, vortexed vigorously, then incubated on ice for 10 minutes. The precipitated protein was pelleted by centrifugation and the supernatant transferred into isopropanol to precipitate the DNA, which was then collected by centrifugation. DNA was washed once in 70% ethanol before air drying and resuspension in the appropriate buffer (either TE buffer or molecular grade water). DNA was run on a 1% agarose gel to confirm no significant degradation had occurred, and stored at 4°C.

### Serial transformation of *S*. *pneumoniae* with genomic DNA

AMX susceptible recipient strains were transformed with 100 μg/mL genomic DNA isolated from AMX resistant strain 11A (Table 1) as above, with the following modifications. DNA uptake occurred during 30 min, followed by recovery for 2 hrs. Transformants were selected by plating inside Columbia agar supplemented with 5% defibrinated sheep blood and a range of AMX (0.03 μg/mL). Ten colonies were then picked at random and streaked onto CBA plates containing AMX. The next day, single colonies were grown up in C+Y with AMX and stocked. MICs were determined for these 10 strains, which were then subjected to another round of transformation and selection on a range of AMX concentrations (0.17–0.5 μg/mL), and 5–10 single colonies were picked for each of the 10 strains transformed. These were isolated, and MICs determined, as above. To test for significant differences between MICs of second-generation strains in different lineages, a Fisher’s Exact Test with Monte Carlo p-value simulation was used.

To control for spontaneous mutations arising during transformation and recombination, cells transformed with either no DNA, or with D39V genomic DNA, were also plated in CBA with AMX (0.03). Neither resulted in visible colonies.

### Whole genome sequencing and quality control

Genomic DNA from recombinant strains from each recipient background, as well as 11A and TIGR4 were Illumina sequenced (PE150, Novogene and Eurofins Genomics), with a mean coverage of 600-fold. Reads were trimmed using Trimmomatic [94], then assembled using SPAdes [95]. Assembly quality was assessed with Quast [96], and read depth was determined in samtools [97]. Previously published D39V Illumina reads were filtered as above and used where necessary (GEO accessions GSE54199 and GSE69729) [98]. Allelic exchange strains (D39V^M53^, D39V^2x38-2b38-M53-1a53^, D39V^1a53^, D39V^2x38-2b38-M95-1a95^) were treated in the same way, then variants were called using bbmap [99], Freebayes [100], and vcftools [101], in order to check for random mutations arising elsewhere in the genome.

11A, TIGR4, and a subset of recombinant strains were sent for PacBio sequencing (Sequel I and II, Lausanne Genomic Technologies Facility). Demultiplexing and quality control were performed using SMRTlink (PacBio), then reads shorter than 1000 bp were removed with filtlong [102]. Recombinant strain reads were mapped to the recipient reference genome using NGMLR and structural variants (SVs) called with sniffles with default settings, except with read mapping quality > 40 and read depth > 50 [103]. Imprecise SVs were then filtered out. DNA methylation detection was performed using motifMaker [104].

Raw read files are deposited with NCBI Sequence Read Archive under BioProject PRJNA789167 (Individual SRA accession numbers for recombinant strains can be found in S4 Table).

### Hybrid assemblies of reference genomes

As PacBio sequencing depth was greater than 200x, genomes were assembled in a long-read only manner using Trycycler [105], followed by short-read polishing.

Briefly, filtered reads were subsampled 12 times and *de novo* assembled in four different assemblers, flye [106], raven [107], wtdbg2 [108], and miniasm plus minipolish [109,110]. Some assemblies were removed during the Trycycler cluster and reconcile steps, leaving the final consensus sequence to be built from seven to eight assemblies. The consensus assembly was subjected to rounds of polishing with PacBio reads using pbmm2 and pbgcpp (PacBio Toolkit), followed by one round of short-read polishing with Pilon [111].

Genomes were annotated in Prokka [112] using Barrnap and Aragorn [113,114] and are deposited at NCBI under BioProject PRJNA789167.

### Recombination detection with NGS

Competitive mapping of filtered illumina reads onto a pseudogenome containing both recipient and donor chromosomes was used for crude visualization of recombination loci (bbmap) [115]. In order to characterize these loci more accurately, filtered reads of recombinant and recipient strains were mapped onto the 11A hybrid genome assembly using smalt, then variants were called with bcftools [116]. SNPs were filtered as described previously [11], and used to build a consensus alignment of donor, recipient, and recombinant strains. The consensus alignment was run through a python script designed to identify SNPs between recombinant and donor which are not shared with the recipient genome. Once identified, the match was extended until the donor and recombinant sequences are no longer identical, then the start, end, and length of detected recombination events are extracted [11].

### Identifying non-contiguous recombination events

Many recombination events were located very close together, with sometimes only a few recipient allele SNPs separating the two, suggesting a single donor DNA molecule as the source. To determine if events were significantly close together, a bootstrapping approach was applied [8]. Firstly, the distances between one recombination event and each of the other recombination events in a single genome were calculated. The circular chromosome gives two values, and the shortest distance between any two events was added to the test population. This was calculated for every sequenced strain in the D39V and TIGR4 recombinants, and for second-generation strains any prior events carried through from the first-generation were removed from the dataset. The shortest recombination distance within a strain became the test value (*d_test_*), and then the test population was sampled (with replacement).

The distribution of a bootstrapped sample (same size as the test population) was used to test the hypothesis that *d_test_* was significantly shorter than expected under the null hypothesis that recombination events are located at random around the chromosome. A Holm correction was applied to account for multiple testing, based on the number of recombination events in the strain. Bootstrap tests were performed one thousand times for each *d_test_* value and considered significantly short if the null hypothesis was rejected at least 95% of the time. If a *d_test_* value was significant, then the distance between the next shortest pairing within the strain became *d_test_*, and was tested in the same fashion. This continued until a *d_test_* where the null hypothesis could not be rejected.

When recombination events were considered significantly close together, they were linked together into non-contiguous recombination events.

### Confirming the origins of non-contiguous recombination events in vivo

A 4.25 Kb region of the 11A genome spanning *pbp2x* was used to test the origin of non-contiguous recombination. This region included an 846 bp stretch with SNPs conferring a small decrease in AMX susceptibility sufficient for selection. One long fragment spanning the entire region was amplified with OVL5375 and OVL5378. Two shorter fragments were then amplified, an upstream fragment (Up) with OVL5379 and OVL5375 (2.84 Kb) and a downstream fragment (Down) with OVL5376 and OVL5378 (2.26 Kb), which overlapped at the SNPs determined to be necessary for selection (S2 and S5 Tables). The three donor DNA templates used in the experiment and their SNPs are shown in Fig 6C (S6 Table).

D39V was transformed as above, with equimolar quantities of each fragment separately, as well as a combination of the two short fragments, and a negative control with no DNA. Transformants were selected on AMX (0.015 μg/mL), and the transformation efficiency was determined by dividing by the total viable count. Individual colonies were restreaked on AMX, then single colonies were picked and the 4.25 Kb region of interest was amplified and Sanger sequenced (Microsynth, S2 Table oligos). Nucleotide sequences were aligned in Mega [117].

As molecule length affects transformation efficiency [80,118,119], an additional experiment was performed, where the amplified fragments were all extended to 6 Kb by the addition of non-homologous DNA amplified from *E*. *coli* and ligated to one end using Golden Gate assembly with BsmBI and T4 DNA ligase (NEB, Vazyme). All oligos and the corresponding templates used in the cloning for these experiments are outlined in S6 Table, and a comprehensive oligo list can be found in S2 Table. The assemblies were then amplified to ensure high quality of transforming fragments, resulting in three fragments of equal size, with differing lengths of homologous regions to the *pbp2x* locus. These were then transformed, amplified, and Sanger sequenced as above, and the transformation efficiency determined.

### Gene and protein alignments

Nucleotide sequences of *pbp* and *murM* genes were translated using Emboss:TransSeq [120]. Alignments were performed with Muscle [121]. Phylogenetic trees were produced using concatenated protein alignments of Pbp2x, Pbp2b, Pbp1a, and MurM as input for FastTree (default settings), and visualised in ITOL [122,123].

### Amplification and transformation of *pbp* and *murM* loci for allelic exchange experiments

Genes for *pbps* were amplified with Phanta Max Super-Fidelity DNA Polymerase (Vazyme) and primers ordered from Sigma (S2 Table). Equimolar quantities of PCR products were used for transformation. To avoid transforming mutations in *murN* alongside those in *murM*, alleles at this locus were cloned into a construct with D39V flanking sequences. The upstream and downstream fragments were amplified from D39V with OVL5540/OVL5779 and OVL5780/OVL5541 respectively, and the *murM* allele from AMR53 or AMR95 with OVL5777/OVL5778. Fragments were then assembled using BsmBI and T4 DNA ligase.

*pbp2x* and *pbp2b* alleles from AMR37 and AMR38 were transformed into D39V as above, selected on 0.012 μg/mL, 0.015 μg/mL, 0.02 μg/mL AMX, and confirmed by Sanger sequencing. D39V^2x38-2b38^ (Table 1) was then used as the recipient for *murM* and *pbp1a* alleles from AMR53 and AMR95, with selection on 0.2 μg/mL, 0.5 μg/mL and 1 μg/mL AMX.

## Supporting information

S1 Table Recombinant strains isolated in sequential rounds of transformation with AMX resistant DNA and their associated MICs. (XLSX)

S2 Table Primers used in this study. (XLSX)

S3 Table List of all recombination events identified in transformants using SNPs. (XLSX)

S4 Table Summary statistics for the characterisation of SNP and recombination results for sequenced recombinant strains. (A) All predicted events detected when compared to parent strain genome. Potential non-contiguity not accounted for. (B) Total DNA transferred during the final round of transformation for that strain. (*) All DNA or SNPs transferred into the original recipient (D39V or TIGR4), through one or two rounds of transformation (depending on the generation). SRA accession numbers for raw Illumina and PacBio reads used for analysis are also provided.
(XLSX)

S5 Table Key amino acid substitutions in PBPs and MurM of recombinant strains. Substitutions listed have been associated with β-lactam resistance in the literature previously. Cells coloured green have the same allele as D39V, those coloured blue have the same allele as 11A. Strain AMX MICs are also indicated for reference.
(XLSX)

S6 Table Cloning design for fragments used to test for single-molecule non-contiguous recombination at the *pbp2x-mraY* locus. (XLSX)

S7 Table Summary statistics from colonies isolated after transformation with different length and combinations of donor fragments to explain the occurrence of non-contiguous recombination. (XLSX)

S8 Table SNPs detected in D39V^M53^. (XLSX)

S1 Fig Fluorescence and coomassie stained images of Bocillin-FL gels. (A) Bocillin-FL labelled cell extracts imaged in an Amersham Typhoon with Cy2 filter setup showing PBP binding affinities, from Fig 3B Coomassie stain of Bocillin-FL gel from Fig 3C Bocillin-FL labelled cell extracts imaged in an Amersham Typhoon Cy2 filter setup from Fig 8D Coomassie stain of Bocillin-FL gel from Fig 8.
(TIF)

S2 Fig Identification of non-contiguous recombination from sequenced recombinant strains using a bootstrapping method (Croucher et al. 2012). (A) Distances between recombination events which were found to shorter than expected under a null hypothesis where events occur randomly around the chromosome. (B) Schematic showing how events were treated if they were found to be closer to each other than expected. (C) Histogram of recombination events length across all sequenced recombinant strains if non-contiguous recombination is not taken into account. (D) Histogram of recombination events length across all sequenced recombinant strains if non-contiguous recombination is accounted for.
(TIF)

S3 Fig Alignments of pbp2x-mraY from colonies isolated after transformation with different length and combinations of donor fragments to explain the occurrence of non-contiguous recombination. Facets show colonies selected from different donor templates which correspond to Fig 6. Bases which match the recipient (D39V) are coloured grey, those which match the donor (11A) are shown in blue. (A) Long, (B) Up, (C) Down.
(TIF)

S4 Fig Alignments of pbp2x-mraY from colonies isolated after transformation with different length and combinations of donor fragments to explain the occurrence of non-contiguous recombination. Facets show colonies selected from different donor templates which correspond to Fig 6. Bases which match the recipient (D39V) are coloured grey, those which match the donor (11A) are shown in blue. (A) Up & Down, (B) Long-NH, (C) NH-Up.
(TIF)

S5 Fig Alignments of pbp2x-mraY from colonies isolated after transformation with different length and combinations of donor fragments to explain the occurrence of non-contiguous recombination. Facets show colonies selected from different donor templates which correspond to Fig 6. Bases which match the recipient (D39V) are coloured grey, those which match the donor (11A) are shown in blue. (A) Down-NH, and (B) NH-Up and Down-NH.
(TIF)

S6 Fig Dendrogram of strain relationships built from amino acid sequences of Pbp and murM proteins. Alignments of Pbp1a, Pbp2b, Pbp2x, and MurM amino acid sequences were used to construct a phylogenetic tree of donor, recipient, and recombinant strains. AMX MICs are shown on the right, color scale represents MIC and corresponds to Fig 2, where yellow is an AMX MIC of 0.01 μg/mL and dark purple is an MIC of 4 μg/mL.
(TIF)

## References

[1] Salvadori G, Junges R, Morrison DA, Petersen FC. Competence in *streptococcus pneumoniae* and close commensal relatives: Mechanisms and implications. Vol. 9, Frontiers in Cellular and Infection Microbiology. Frontiers; 2019. p. 94. doi: 10.3389/fcimb.2019.00094 31001492 PMC6456647

[2] O’Brien KL, Wolfson LJ, Watt JP, Henkle E, Deloria-Knoll M, McCall N, et al. Burden of disease caused by *Streptococcus pneumoniae* in children younger than 5 years: global estimates. The Lancet. 2009 Sep 12;374(9693):893–902. Available from: https://www.sciencedirect.com/science/article/pii/S0140673609612046. 19748398 10.1016/S0140-6736(09)61204-6

[3] Shak JR, Vidal JE, Klugman KP. Influence of bacterial interactions on pneumococcal colonization of the nasopharynx. Trends Microbiol. 2013 Mar:129–35. Available from: http://www.ncbi.nlm.nih.gov/pubmed/23273566. 23273566 10.1016/j.tim.2012.11.005 PMC3729046

[4] Croucher NJ, Harris SR, Fraser C, Quail MA, Burton J, Van Der Linden M, et al. Rapid pneumococcal evolution in response to clinical interventions. Science (1979). 2011 Jan 28;331(6016):430–4. doi: 10.1126/science.1198545 21273480 PMC3648787

[5] Wyres KL, Lambertsen LM, Croucher NJ, McGee L, Von Gottberg A, Liñares J, et al. Pneumococcal capsular switching: A historical perspective. Journal of Infectious Diseases. 2013;207(3):439–49. doi: 10.1093/infdis/jis703 23175765 PMC3537446

[6] Dowson CG, Coffey TJ, Kell C, Whiley RA. Evolution of penicillin resistance in *Streptococcus pneumoniae*; the role of *Streptococcus* mitis in the formation of a low affinity PBP2B in *S. pneumoniae*. Molecular Microbiology. 1993 Aug 1;9(3):635–43. Available from: https://onlinelibrary.wiley.com/doi/full/10.1111/j.1365-2958.1993.tb01723.x. 8412708 10.1111/j.1365-2958.1993.tb01723.x

[7] Sibold C, Henrichsen J, König A, Martin C, Chalkley L, Hakenbeck R. Mosaic pbpX genes of major clones of penicillin-resistant *Streptococcus pneumoniae* have evolved from pbpX genes of a penicillin-sensitive *Streptococcus oralis*. Molecular Microbiology. 1994 Jun 1;12(6):1013–23. Available from: https://onlinelibrary.wiley.com/doi/full/10.1111/j.1365-2958.1994.tb01089.x. 7934893 10.1111/j.1365-2958.1994.tb01089.x

[8] Croucher NJ, Harris SR, Barquist L, Parkhill J, Bentley SD. A High-Resolution View of Genome-Wide Pneumococcal Transformation. Didelot X, editor. PLoS Pathogens. 2012 Jun 14;8(6):e1002745. Available from: https://dx.plos.org/10.1371/journal.ppat.1002745. 22719250 10.1371/journal.ppat.1002745 PMC3375284

[9] Hiller NL, Ahmed A, Powell E, Martin DP, Eutsey R, Earl J, et al. Generation of Genetic Diversity among *Streptococcus pneumoniae* Strains via Horizontal Gene Transfer during a Chronic Polyclonal Pediatric Infection. PLOS Pathogens. 2010 Sep;6(9):e1001108. Available from: https://journals.plos.org/plospathogens/article?id=10.1371/journal.ppat.1001108. 20862314 10.1371/journal.ppat.1001108 PMC2940740

[10] Gurney T, Fox MS. Physical and genetic hybrids formed in bacterial transformation. Journal of Molecular Biology. 1968;32(1):83–100. doi: 10.1016/0022-2836(68)90147-2 4384454

[11] Cowley LA, Petersen FC, Junges R, Jimson D. Jimenez M, Morrison DA, Hanage WP. Evolution via recombination: Cell-to-cell contact facilitates larger recombination events in *Streptococcus pneumoniae*. Matic, editor. PLOS Genetics. 2018 Jun 13;14(6):e1007410. Available from: http://dx.plos.org/10.1371/journal.pgen.1007410. 29897968 10.1371/journal.pgen.1007410 PMC6016952

[12] Wyres KL, Lambertsen LM, Croucher NJ, McGee L, von Gottberg A, Liñares J, et al. The multidrug-resistant PMEN1 pneumococcus is a paradigm for genetic success. Genome Biology. 2012;13(11):R103. Available from: http://genomebiology.com/2012/13/11/R103. 23158461 10.1186/gb-2012-13-11-r103 PMC3580495

[13] Donati C, Hiller NL, Tettelin H, Muzzi A, Croucher NJ, Angiuoli S V., et al. Structure and dynamics of the pan-genome of *Streptococcus pneumoniae* and closely related species. Genome Biology. 2010 Oct 29;11(10):R107. Available from: https://genomebiology.biomedcentral.com/articles/10.1186/gb-2010-11-10-r107. 21034474 10.1186/gb-2010-11-10-r107 PMC3218663

[14] Straume D, Stamsås GA, Håvarstein LS. Natural transformation and genome evolution in *Streptococcus pneumoniae*. Vol. 33, Infection, Genetics and Evolution. Elsevier; 2015. p. 371–80. doi: 10.1016/j.meegid.2014.10.020 25445643

[15] Ween O, Gaustad P, Håvarstein LS. Identification of DNA binding sites for ComE, a key regulator of natural competence in *Streptococcus pneumoniae*. Molecular Microbiology. 1999 Aug 1;33(4):817–27. Available from: https://onlinelibrary.wiley.com/doi/full/10.1046/j.1365-2958.1999.01528.x. 10447890 10.1046/j.1365-2958.1999.01528.x

[16] Martin B, Soulet AL, Mirouze N, Prudhomme M, Mortier-Barrière I, Granadel C, et al. ComE/ComE∼P interplay dictates activation or extinction status of pneumococcal X-state (competence). Molecular Microbiology. 2013 Jan 1;87(2):394–411. Available from: https://onlinelibrary.wiley.com/doi/full/10.1111/mmi.12104. 23216914 10.1111/mmi.12104

[17] Slager J, Aprianto R, Veening JW. Refining the Pneumococcal Competence Regulon by RNA Sequencing. J Bacteriol. 2019 Jul 1;201(13):e00780–18. Available from: http://www.ncbi.nlm.nih.gov/pubmed/30885934. 30885934 10.1128/JB.00780-18 PMC6560143

[18] Rosenthal AL, Lacks SA. Complex structure of the membrane nuclease of *Streptococcus pneumoniae* revealed by two-dimensional electrophoresis. Journal of Molecular Biology. 1980;141(2):133–46. doi: 10.1016/0022-2836(80)90381-2 6255165

[19] Lacks S, Neuberger M. Membrane location of a deoxyribonuclease implicated in the genetic transformation of Diplococcus pneumoniae. Journal of Bacteriology. 1975;124(3):1321–9. doi: 10.1128/jb.124.3.1321-1329.1975 366 PMC236044

[20] Lacks S, Greenberg B. Single-strand breakage on binding of DNA to cells in the genetic transformation of Diplococcus pneumoniae. Journal of Molecular Biology. 1976;101(2):255–75. doi: 10.1016/0022-2836(76)90376-4 4625

[21] Bergé MJ, Kamgoué A, Martin B, Polard P, Campo N, Claverys JP. Midcell Recruitment of the DNA Uptake and Virulence Nuclease, EndA, for Pneumococcal Transformation. PLOS Pathogens. 2013 Sep;9(9):e1003596. Available from: https://journals.plos.org/plospathogens/article?id=10.1371/journal.ppat.1003596. 24039578 10.1371/journal.ppat.1003596 PMC3764208

[22] Laurenceau R, Péhau-Arnaudet G, Baconnais S, Gault J, Malosse C, Dujeancourt A, et al. A Type IV Pilus Mediates DNA Binding during Natural Transformation in *Streptococcus pneumoniae*. PLoS Pathogens. 2013 Jun;9(6):e1003473. Available from: https://journals.plos.org/plospathogens/article?id=10.1371/journal.ppat.1003473. 23825953 10.1371/journal.ppat.1003473 PMC3694846

[23] Attaiech L, Olivier A, Mortier-Barrière I, Soulet AL, Granadel C, Martin B, et al. Role of the single-stranded dna-binding protein Ssbb in pneumococcal transformation: Maintenance of a reservoir for genetic plasticity. PLoS Genetics. 2011 Jun;7(6):e1002156. Available from: https://journals.plos.org/plosgenetics/article?id=10.1371/journal.pgen.1002156. 21738490 10.1371/journal.pgen.1002156 PMC3128108

[24] Morrison DA, Mortier-Barrière I, Attaiech L, Claverys JP. Identification of the major protein component of the pneumococcal eclipse complex. Journal of Bacteriology. 2007 Sep;189(17):6497–500. Available from: https://journals.asm.org/journal/jb. 17601792 10.1128/JB.00687-07 PMC1951911

[25] Lisboa J, Andreani J, Sanchez D, Boudes M, Collinet B, Liger D, et al. Molecular determinants of the DprA−RecA interaction for nucleation on ssDNA. Nucleic Acids Research. 2014 Jun 17;42(11):7395–408. Available from: https://academic.oup.com/nar/article/42/11/7395/1442014. 24782530 10.1093/nar/gku349 PMC4066776

[26] Mortier-Barrière I, Velten M, Dupaigne P, Mirouze N, Piétrement O, McGovern S, et al. A Key Presynaptic Role in Transformation for a Widespread Bacterial Protein: DprA Conveys Incoming ssDNA to RecA. Cell. 2007 Sep 7;130(5):824–36. doi: 10.1016/j.cell.2007.07.038 17803906

[27] Quevillon-Cheruel S, Campo N, Mirouze N, Mortier-Barrière I, Brooks MA, Boudes M, et al. Structure-function analysis of pneumococcal DprA protein reveals that dimerization is crucial for loading RecA recombinase onto DNA during transformation. Proc Natl Acad Sci U S A. 2012 Sep 11;109(37):E2466–75. Available from: https://www.pnas.org/content/109/37/E2466. 22904190 10.1073/pnas.1205638109 PMC3443122

[28] Mirouze N, Bergé MA, Soulet AL, Mortier-Barrière I, Quentin Y, Fichant G, et al. Direct involvement of DprA, the transformation-dedicated RecA loader, in the shut-off of pneumococcal competence. Proc Natl Acad Sci USA. 2013;110(11). Available from: www.pnas.org/cgi/doi/10.1073/pnas.1219868110. 23440217 10.1073/pnas.1219868110 PMC3600483

[29] Piotrowski A, Luo P, Morrison DA. Competence for Genetic Transformation in *Streptococcus pneumoniae*: Termination of Activity of the Alternative Sigma Factor ComX Is Independent of Proteolysis of ComX and ComW. Journal of Bacteriology. 2009 May;191(10):3359. Available from: /pmc/articles/PMC2687157/. doi: 10.1128/JB.01750-08 19286798 PMC2687157

[30] Weng L, Piotrowski A, Morrison DA. Exit from Competence for Genetic Transformation in *Streptococcus pneumoniae* Is Regulated at Multiple Levels. PLoS ONE. 2013 May 22;8(5):e64197. Available from: https://journals.plos.org/plosone/article?id=10.1371/journal.pone.0064197. 23717566 10.1371/journal.pone.0064197 PMC3661451

[31] Cassone M, Gagne AL, Spruce LA, Seeholzer SH, Sebert ME. The HtrA protease from *Streptococcus pneumoniae* digests both denatured proteins and the competence-stimulating peptide. J Biol Chem. 2012 Nov 9;287(46):38449–59. Available from: http://www.ncbi.nlm.nih.gov/pubmed/23012372. 23012372 10.1074/jbc.M112.391482 PMC3493890

[32] Alloing G, Martin B, Granadel C, Claverys JP. Development of competence in *Streptococcus pneumoniae*: Pheromone autoinduction and control of quorum sensing by the oligopeptide permease. Molecular Microbiology. 1998;29(1):75–83. doi: 10.1046/j.1365-2958.1998.00904.x 9701804

[33] Forget AL, Kowalczykowski SC. Single-molecule imaging of DNA pairing by RecA reveals a three-dimensional homology search. Nature 2012 482:7385. 2012 Feb 8;482(7385):423–7. Available from: https://www.nature.com/articles/nature10782. 22318518 10.1038/nature10782 PMC3288143

[34] Yang H, Zhou C, Dhar A, Pavletich NP. Mechanism of strand exchange from RecA–DNA synaptic and D-loop structures. Nature. 2020;586(7831):801–6. Available from: doi: 10.1038/s41586-020-2820-9 33057191 PMC8366275

[35] Wiktor J, Gynnå AH, Leroy P, Larsson J, Coceano G, Testa I, et al. RecA finds homologous DNA by reduced dimensionality search. Nature. 2021 Sep 1;597(7876):426–9. Available from: https://www.nature.com/articles/s41586-021-03877-6. 34471288 10.1038/s41586-021-03877-6 PMC8443446

[36] Bell JC, Kowalczykowski SC. RecA: Regulation and Mechanism of a Molecular Search Engine. Vol. 41, Trends in Biochemical Sciences. 2016. p. 491–507. Available from: https://reader.elsevier.com/reader/sd/pii/S0968000416300056?token=47B3E2CB5DCF3AD8B1BFE0623FE6C21F0F109D04126C00002EEA1583F52F5E30433559D1D7B07483293062C38D963C94&originRegion=eu-west-1&originCreation=20210928112241. 27156117 10.1016/j.tibs.2016.04.002 PMC4892382

[37] Torres R, Serrano E, Alonso JC. Bacillus subtilis RecA interacts with and loads RadA/Sms to unwind recombination intermediates during natural chromosomal transformation. Nucleic Acids Research. 2019 Sep 26;47(17):9198–215. Available from: https://academic.oup.com/nar/article/47/17/9198/5539883. 31350886 10.1093/nar/gkz647 PMC6755099

[38] Hsieh P, Camerini-Otero CS, Camerini-Otero RD. The synapsis event in the homologous pairing of DNAs: RecA recognizes and pairs less than one helical repeat of DNA. Proc Natl Acad Sci U S A. 1992;89(14):6492–6. doi: 10.1073/pnas.89.14.6492 1631148 PMC49527

[39] Mazin A V, Kowalczykowski SC. The specificity of the secondary DNA binding site of RecA protein defines its role in DNA strand exchange. Proc Natl Acad Sci U S A. 1996;93(20):10673–8. doi: 10.1073/pnas.93.20.10673 8855238 PMC38213

[40] Marie L, Rapisarda C, Morales V, Bergé M, Perry T, Soulet AL, et al. Bacterial RadA is a DnaB-Type helicase interacting with RecA to promote bidirectional D-loop extension. Nature Communications. 2017 May 31;8(1):1–14. Available from: https://www.nature.com/articles/ncomms15638. 10.1038/ncomms15638 PMC5512693 28561029

[41] Desai B V, Morrison DA. Transformation in *Streptococcus pneumoniae*: formation of eclipse complex in a coiA mutant implicates CoiA in genetic recombination. Molecular Microbiology. 2007;63(4):1107–17. doi: 10.1111/j.1365-2958.2006.05558.x 17233830

[42] Danilowicz C, Yang D, Kelley C, Prévost C, Prévost P, Prentiss M. The poor homology stringency in the heteroduplex allows strand exchange to incorporate desirable mismatches without sacrificing recognition in vivo. Nucleic Acids Research. 2015;43(13):6473–85. Available from: https://academic.oup.com/nar/article/43/13/6473/2414306. 26089391 10.1093/nar/gkv610 PMC4513875

[43] Carrasco B, Serrano E, Sánchez H, Wyman C, Alonso JC. Chromosomal transformation in Bacillus subtilis is a non-polar recombination reaction. Nucleic Acids Research. 2016 Apr 7;44(6):2754–68. Available from: https://academic.oup.com/nar/article/44/6/2754/2499447. 26786319 10.1093/nar/gkv1546 PMC4824099

[44] Claverys JP, Lacks SA. Heteroduplex deoxyribonucleic acid base mismatch repair in bacteria. Vol. 50, Microbiological Reviews. American Society for Microbiology (ASM); 1986. p. 133–65. Available from: https://www.ncbi.nlm.nih.gov/pmc/articles/PMC373061/. 3523187 10.1128/mr.50.2.133-165.1986 PMC373061

[45] Gasc AM, Sicard AM, Claverys JP. Repair of single- and multiple-substitution mismatches during recombination in *Streptococcus pneumoniae*. Genetics. 1989;121(1):29–36. doi: 10.1093/genetics/121.1.29 2645195 PMC1203602

[46] Humbert O, Prudhomme M, Hakenbeck R, Dowson CG, Claverys JP. Homeologous recombination and mismatch repair during transformation in *Streptococcus pneumoniae*: Saturation of the Hex mismatch repair system. Proc Natl Acad Sci USA. 1995;92(20):9052–6. Available from: https://www.jstor.org/stable/2368408?seq=1#metadata_info_tab_contents. 7568071 10.1073/pnas.92.20.9052 PMC40922

[47] Scheffers DJ, Pinho MG. Bacterial Cell Wall Synthesis: New Insights from Localization Studies. Microbiology and Molecular Biology Reviews. 2005 Dec;69(4):585–607. Available from: https://pubmed.ncbi.nlm.nih.gov/16339737/. 16339737 10.1128/MMBR.69.4.585-607.2005 PMC1306805

[48] Hakenbeck R, Brückner R, Denapaite D, Maurer P. Molecular mechanisms of β-lactam resistance in *Streptococcus pneumoniae*. Vol. 7, Future Microbiology. Future Medicine Ltd London, UK; 2012. p. 395–410. Available from: https://www.futuremedicine.com/doi/10.2217/fmb.12.2. 22393892 10.2217/fmb.12.2

[49] Kalizang’oma A, Chaguza C, Gori A, Davison C, Beleza S, Antonio M, et al. *Streptococcus pneumoniae* serotypes that frequently colonise the human nasopharynx are common recipients of penicillin-binding protein gene fragments from *Streptococcus mitis*. Microbial Genomics. 2021 Sep 22;7(9):000622. Available from: https://pubmed.ncbi.nlm.nih.gov/34550067/. doi: 10.1099/mgen.0.000622 34550067 PMC8715442

[50] Doern G V, Brueggemann A, Holley HP, Rauch AM. Antimicrobial resistance of *Streptococcus pneumoniae* recovered from outpatients in the United States during the winter months of 1994 to 1995: results of a 30-center national surveillance study. Antimicrobial Agents Chemotherapy. 1996 May 1;40(5):1208–13. Available from: http://www.ncbi.nlm.nih.gov/pubmed/8723468. doi: 10.1128/AAC.40.5.1208 8723468 PMC163293

[51] Doit C, Loukil C, Fitoussi F, Geslin P, Bingen E. Emergence in France of Multiple Clones of Clinical *Streptococcus pneumoniae* Isolates with High-Level Resistance to Amoxicillin. Antimicrobial Agents and Chemotherapy. 1999 Jun 1;43(6):1480–3. Available from: http://www.ncbi.nlm.nih.gov/pubmed/10348775. doi: 10.1128/AAC.43.6.1480 10348775 PMC89301

[52] Càmara J, Cubero M, Martín-Galiano AJ, García E, Grau I, Nielsen JB, et al. Evolution of the β-lactam-resistant *Streptococcus pneumoniae* PMEN3 clone over a 30 year period in Barcelona, Spain. Journal of Antimicrobial Chemotherapy. 2018;73(11):2941–51. Available from: https://academic.oup.com/jac/advance-article/doi/10.1093/jac/dky305/5079858. 30165641 10.1093/jac/dky305

[53] Stanhope MJ, Walsh SL, Becker JA, Miller LA, Lefébure T, Lang P, et al. The relative frequency of intraspecific lateral gene transfer of penicillin binding proteins 1a, 2b, and 2x, in amoxicillin resistant *Streptococcus pneumoniae*. Infection, Genetics and Evolution. 2007 Jul 1;7(4):520–34. Available from: https://www.sciencedirect.com/science/article/pii/S1567134807000408. doi: 10.1016/j.meegid.2007.03.004 17475572

[54] Cafini F, del Campo R, Alou L, Sevillano D, Morosini MI, Baquero F, et al. Alterations of the penicillin-binding proteins and murM alleles of clinical *Streptococcus pneumoniae* isolates with high-level resistance to amoxicillin in Spain. Journal of Antimicrobial Chemotherapy. 2006 Feb 1;57(2):224–9. Available from: http://academic.oup.com/jac/article/57/2/224/804622/Alterations-of-the-penicillinbinding-proteins-and. doi: 10.1093/jac/dki442 16368701

[55] Kosowska K, Jacobs MR, Bajaksouzian S, Koeth L, Appelbaum PC. Alterations of Penicillin-Binding Proteins 1A, 2X, and 2B in *Streptococcus pneumoniae* Isolates for Which Amoxicillin MICs Are Higher than Penicillin MICs. 2004 Oct 1;48(10). Available from: http://www.ncbi.nlm.nih.gov/pubmed/15388470. doi: 10.1128/AAC.48.10.4020-4022.2004 15388470 PMC521876

[56] du Plessis M, Bingen E, Klugman KP, Plessis M du, Bingen E, Klugman KP, et al. Analysis of Penicillin-Binding Protein Genes of Clinical Isolates of *Streptococcus pneumoniae* with Reduced Susceptibility to Amoxicillin. Antimicrobial Agents and Chemotherapy. 2002 Aug 1;46(8):2349–57. Available from: http://www.ncbi.nlm.nih.gov/pubmed/12121904. doi: 10.1128/AAC.46.8.2349-2357.2002 12121904 PMC127354

[57] Chesnel L, Carapito R, Croizé J, Dideberg O, Vernet T, Zapun A. Identical penicillin-binding domains in penicillin-binding proteins of *Streptococcus pneumoniae* clinical isolates with different levels of beta-lactam resistance. Antimicrob Agents Chemother. 2005 Jul 1;49(7):2895–902. Available from: http://www.ncbi.nlm.nih.gov/pubmed/15980366. doi: 10.1128/AAC.49.7.2895-2902.2005 15980366 PMC1168675

[58] Hakenbeck R, Nig AK, Kern I, Van Der Linden M, Keck W, Billot-Klein D, et al. Acquisition of Five High-M Penicillin-Binding Protein Variants during Transfer of High-Level β-Lactam Resistance from *Streptococcus mitis* to *Streptococcus pneumoniae*. Journal of Bacteriology. 1998;180(7):1831–40. doi: 10.1128/JB.180.7.1831-1840.1998 9537382 PMC107097

[59] Albarracín Orio AG, Piñas GE, Cortes PR, Cian MB, Echenique J. Compensatory Evolution of pbp Mutations Restores the Fitness Cost Imposed by β-Lactam Resistance in *Streptococcus pneumoniae*. Levin B, editor. PLoS Pathogens. 2011 Feb 17;7(2):e1002000. Available from: http://dx.plos.org/10.1371/journal.ppat.1002000. 21379570 10.1371/journal.ppat.1002000 PMC3040684

[60] Garcia-Bustos J, Tomasz A. A biological price of antibiotic resistance: major changes in the peptidoglycan structure of penicillin-resistant pneumococci. Proc Natl Acad Sci USA. 1990 Jul 1;87(14):5415–9. Available from: http://www.ncbi.nlm.nih.gov/pubmed/2371278. doi: 10.1073/pnas.87.14.5415 2371278 PMC54335

[61] Smith AM, Klugman KP. Alterations in MurM, a cell wall muropeptide branching enzyme, increase high-level penicillin and cephalosporin resistance in *Streptococcus pneumoniae*. Antimicrobial Agents and Chemotherapy. 2001 Aug 1;45(8):2393–6. Available from: https://aac.asm.org/content/45/8/2393.short. doi: 10.1128/AAC.45.8.2393-2396.2001 11451707 PMC90664

[62] Filipe SR, Tomasz A. Inhibition of the expression of penicillin resistance in *Streptococcus pneumoniae* by inactivation of cell wall muropeptide branching genes. Proceedings of the National Academy of Sciences. 2000;97(9):4891–6. doi: 10.1073/pnas.080067697 10759563 PMC18328

[63] Filipe SR, Severina E, Tomasz A. The murMN operon: A functional link between antibiotic resistance and antibiotic tolerance in *Streptococcus pneumoniae*. Proceedings of the National Academy of Sciences. 2002 Feb 5;99(3):1550–5. Available from: https://www.pnas.org/content/99/3/1550.short. 10.1073/pnas.032671699 PMC122228 11830670

[64] Berg KH, Stamsås GA, Straume D, Håvarstein LS. Effects of low PBP2b levels on cell morphology and peptidoglycan composition in *Streptococcus pneumoniae* R6. Journal of Bacteriology. 2013;195(19):4342–54. Available from: https://journals.asm.org/doi/abs/10.1128/JB.00184-13. 23873916 10.1128/JB.00184-13 PMC3807472

[65] Grebe T, Paik J, Hakenbeck R. A novel resistance mechanism against β-lactams in *Streptococcus pneumoniae* involves CpoA, a putative glycosyltransferase. Journal of Bacteriology. 1997 May 1;179(10):3342–9. Available from: http://www.ncbi.nlm.nih.gov/pubmed/9150233. doi: 10.1128/jb.179.10.3342-3349.1997 9150233 PMC179116

[66] Guenzi E, Gasc AMA-M, Sicard MA, Hakenbeck R. A two-component signal-transducing system is involved in competence and penicillin susceptibility in laboratory mutants of *Streptococcus pneumoniae*. Molecular Microbiology. 1994 May 1;12(3):505–15. Available from: http://doi.wiley.com/10.1111/j.1365-2958.1994.tb01038.x. 8065267 10.1111/j.1365-2958.1994.tb01038.x

[67] Stanhope MJ, Lefébure T, Walsh SL, Becker JA, Lang P, Pavinski Bitar PD, et al. Positive selection in penicillin-binding proteins 1a, 2b, and 2x from *Streptococcus pneumoniae* and its correlation with amoxicillin resistance development. Infection, Genetics and Evolution. 2008 May 1;8(3):331–9. Available from: https://www.sciencedirect.com/science/article/pii/S1567134808000154. doi: 10.1016/j.meegid.2008.02.001 18394970

[68] Arnold BJ, Gutmann MU, Grad YH, Sheppard SK, Corander J, Lipsitch M, et al. Weak epistasis may drive adaptation in recombining bacteria. Genetics. 2018;208(3):1247–60. Available from: 10.1534/genetics.117.300662. 29330348 PMC5844334

[69] Kulick S, Moccia C, Didelot X, Falush D, Kraft C, Suerbaum S. Mosaic DNA Imports with Interspersions of Recipient Sequence after Natural Transformation of Helicobacter pylori. PLOS ONE. 2008 Nov 24;3(11):e3797. Available from: https://journals.plos.org/plosone/article?id=10.1371/journal.pone.0003797. 19030104 10.1371/journal.pone.0003797 PMC2582958

[70] Mell JC, Shumilina S, Hall IM, Redfield RJ. Transformation of natural genetic variation into *haemophilus influenzae* genomes. Guttman DS, editor. PLoS Pathogens. 2011 Jul 28;7(7):e1002151. Available from: https://dx.plos.org/10.1371/journal.ppat.1002151. 21829353 10.1371/journal.ppat.1002151 PMC3145789

[71] Zhao G, Meier TI, Kahl SD, Gee KR, Blaszczak LC. BOCILLIN FL, a sensitive and commercially available reagent for detection of penicillin-binding proteins. Antimicrobial Agents and Chemotherapy. 1999;43(5):1124–8. Available from: https://journals.asm.org/doi/abs/10.1128/AAC.43.5.1124. 10223924 10.1128/aac.43.5.1124 PMC89121

[72] Kocaoglu O, Tsui HCCT, Winkler ME, Carlson EE. Profiling of β-lactam selectivity for penicillin-binding proteins in *Streptococcus pneumoniae* D39. Antimicrobial Agents and Chemotherapy. 2015 Jun 1;59(6):3548–55. Available from: http://www.ncbi.nlm.nih.gov/pubmed/25845878. doi: 10.1128/AAC.05142-14 25845878 PMC4432181

[73] Slager J, Aprianto R, Veening JW. Deep genome annotation of the opportunistic human pathogen *Streptococcus pneumoniae* D39. Nucleic Acids Research. 2018;46(19):9971–89. Available from: /pmc/articles/PMC6212727/. doi: 10.1093/nar/gky725 30107613 PMC6212727

[74] Liu X, Li JW, Feng Z, Luo Y, Veening JW, Zhang JR. Transcriptional repressor PtvR regulates phenotypic tolerance to vancomycin in *Streptococcus pneumoniae*. Journal of Bacteriology. 2017 Jul 1;199(14):54–71. Available from: https://journals.asm.org/doi/abs/10.1128/JB.00054-17. 28484041 10.1128/JB.00054-17 PMC5494751

[75] Münch D, Roemer T, Lee SH, Engeser M, Sahl HG, Schneider T. Identification and in vitro Analysis of the GatD/MurT Enzyme-Complex Catalyzing Lipid II Amidation in *Staphylococcus aureus*. PLOS Pathogens. 2012 Jan;8(1):e1002509. Available from: https://journals.plos.org/plospathogens/article?id=10.1371/journal.ppat.1002509. 22291598 10.1371/journal.ppat.1002509 PMC3266927

[76] Figueiredo TA, Sobral RG, Ludovice AM, de Almeida JMF, Bui NK, Vollmer W, et al. Identification of Genetic Determinants and Enzymes Involved with the Amidation of Glutamic Acid Residues in the Peptidoglycan of *Staphylococcus aureus*. PLOS Pathogens. 2012 Jan;8(1):e1002508. Available from: https://journals.plos.org/plospathogens/article?id=10.1371/journal.ppat.1002508. 22303291 10.1371/journal.ppat.1002508 PMC3267633

[77] Manso AS, Chai MH, Atack JM, Furi L, De Ste Croix M, Haigh R, et al. A random six-phase switch regulates pneumococcal virulence via global epigenetic changes. Nature Communications. 2014 Sep 30;5(1):1–9. Available from: https://www.nature.com/articles/ncomms6055. doi: 10.1038/ncomms6055 25268848 PMC4190663

[78] Feng Z, Li J, Zhang JR, Zhang X. Qdnamod: A statistical model-based tool to reveal intercellular heterogeneity of DNA modification from SMRT sequencing data. Nucleic Acids Research. 2014;42(22):13488–99. Available from: https://academic.oup.com/nar/article/42/22/13488/2411011. doi: 10.1093/nar/gku1097 25404133 PMC4267614

[79] Mortier-Barriere I, Humbert O, Martin B, Prudhomme M, Claverys JP. Control of recombination rate during transformation of *Streptococcus pneumoniae*: An overview. Microbial Drug Resistance. 1997 Jan 29;3(3):233–42. doi: 10.1089/mdr.1997.3.233 9270992

[80] Kurushima J, Campo N, van Raaphorst R, Cerckel G, Polard P, Veening JW. Unbiased homeologous recombination during pneumococcal transformation allows for multiple chromosomal integration events. Elife. 2020 Sep 1;9:1–70. doi: 10.7554/eLife.58771 32965219 PMC7567608

[81] Morrison DA, Guild WR. Transformation and deoxyribonucleic acid size: extent of degradation on entry varies with size of donor. J Bacteriol. 1972;112(3):1157–68. Available from: https://journals.asm.org/journal/jb. doi: 10.1128/jb.112.3.1157-1168.1972 4404818 PMC251544

[82] Morrison DA, Guild WR. Breakage prior to entry of donor DNA in Pneumococcus transformation. BBA Section Nucleic Acids and Protein Synthesis. 1973 Apr 11;299(4):545–56. doi: 10.1016/0005-2787(73)90226-8 4145314

[83] El Khoury JY, Boucher N, Bergeron MG, Leprohon P, Ouellette M. Penicillin induces alterations in glutamine metabolism in *Streptococcus pneumoniae*. Scientific Reports. 2017 Dec 1;7(1). Available from: /pmc/articles/PMC5673960/. doi: 10.1038/s41598-017-15035-y 29109543 PMC5673960

[84] Chewapreecha C, Harris SR, Croucher NJ, Turner C, Marttinen P, Cheng L, et al. Dense genomic sampling identifies highways of pneumococcal recombination. Nature Genetics. 2014 Feb 9;46(3):305–9. Available from: https://www.nature.com/articles/ng.2895. doi: 10.1038/ng.2895 24509479 PMC3970364

[85] Trzciński K, Thompson CM, Lipsitch M. Single-step capsular transformation and acquisition of penicillin resistance in *Streptococcus pneumoniae*. Journal of Bacteriology. 2004 Jun;186(11):3447–52. Available from: http://www.mlst.net. doi: 10.1128/JB.186.11.3447-3452.2004 15150231 PMC415782

[86] Fani F, Leprohon P, Zhanel GG, Bergeron MG, Ouellette M. Genomic analyses of DNA transformation and penicillin resistance in *Streptococcus pneumoniae* clinical isolates. Antimicrobial Agents and Chemotherapy. 2014 Mar 16;58(3):1397–403. Available from: https://journals.asm.org/doi/full/10.1128/AAC.01311-13. 24342643 10.1128/AAC.01311-13 PMC3957846

[87] Shockley TE, Hotchkiss RD. Stepwise introduction of transformable penicillin resistance in Pneumococcus. Genetics. 1970;64(3):397–408. 4393537 PMC1212409

[88] Hotchkiss RD. Transfer of penicillin resistance in pneumococci by the desoxyribonucleate derived from resistant cultures. Cold Spring Harb Symp Quant Biol. 1951 Jan 1;16:457–61. Available from: http://symposium.cshlp.org/content/16/457. doi: 10.1101/sqb.1951.016.01.032 14942755

[89] Demerec M. Production of Staphylococcus strains resistant to various concentrations of penicillin. Proceedings of the National Academy of Sciences. 1945;31(1):16–24. doi: 10.1073/pnas.31.1.16 16588677 PMC1078743

[90] Johnsborg O, Eldholm V, Bjørnstad ML, Håvarstein LS. A predatory mechanism dramatically increases the efficiency of lateral gene transfer in *Streptococcus pneumoniae* and related commensal species. Molecular Microbiology. 2008 Jul 1;69(1):245–53. Available from: http://doi.wiley.com/10.1111/j.1365-2958.2008.06288.x. 18485065 10.1111/j.1365-2958.2008.06288.x

[91] Eldholm V, Johnsborg O, Haugen K, Ohnstad HS, Havastein LS. Fratricide in *Streptococcus pneumoniae*: Contributions and role of the cell wall hydrolases CbpD, LytA and LytC. Microbiology (N Y). 2009;155(7):2223–34. doi: 10.1099/mic.0.026328-0 19389766

[92] Martin B, Garcia P, Castanié M-P, Claverys J-P. The recA gene of *Streptococcus pneumoniae* is part of a competence-induced operon and controls lysogenic induction. Molecular Microbiology. 1995 Jan 1;15(2):367–79. doi: 10.1111/j.1365-2958.1995.tb02250.x 7538190

[93] Charles MK, Berenger BM, Turnbull L, Rennie R, Fuller J. Variability of β-lactam susceptibility testing for *Streptococcus pneumoniae* using 4 commercial test methods and broth microdilution. Diagnostic Microbiology and Infectious Disease. 2016 Mar 1;84(3):240–5. Available from: https://www.sciencedirect.com/science/article/pii/S0732889315004137. doi: 10.1016/j.diagmicrobio.2015.11.014 26707068

[94] Bolger AM, Lohse M, Usadel B. Trimmomatic: a flexible trimmer for Illumina sequence data. Bioinformatics. 2014 Aug 1;30(15):2114–20. Available from: http://www.ncbi.nlm.nih.gov/pubmed/24695404. doi: 10.1093/bioinformatics/btu170 24695404 PMC4103590

[95] Nurk S, Bankevich A, Antipov D, Gurevich A, Korobeynikov A, Lapidus A, et al. Assembling Genomes and Mini-metagenomes from Highly Chimeric Reads. In: In: Deng M, Jiang R, Sun F, Zhang X (eds) Research in Computational Molecular Biology. Springer, Berlin, Heidelberg; 2013. p. 158–70. Available from: http://link.springer.com/10.1007/978-3-642-37195-0_13.

[96] Gurevich A, Saveliev V, Vyahhi N, Tesler G. QUAST: quality assessment tool for genome assemblies. Bioinformatics. 2013 Apr 15;29(8):1072–5. Available from: https://academic.oup.com/bioinformatics/article-lookup/doi/10.1093/bioinformatics/btt086. 23422339 10.1093/bioinformatics/btt086 PMC3624806

[97] Li H, Handsaker B, Wysoker A, Fennell T, Ruan J, Homer N, et al. The Sequence Alignment/Map format and SAMtools. Bioinformatics. 2009 Aug 15;25(16):2078–9. Available from: http://www.ncbi.nlm.nih.gov/pubmed/19505943. doi: 10.1093/bioinformatics/btp352 19505943 PMC2723002

[98] Slager J, Kjos M, Attaiech L, Veening JW. Antibiotic-induced replication stress triggers bacterial competence by increasing gene dosage near the origin. Cell. 2014;157(2):395–406. Available from: doi: 10.1016/j.cell.2014.01.068 24725406

[99] Bushnell B. BBMap download | SourceForge.net. SourceForge. 2013. Available from: https://sourceforge.net/projects/bbmap/.

[100] Garrison E, Marth G. Haplotype-based variant detection from short-read sequencing. arXiv.org. 2012 Jul 17; Available from: http://arxiv.org/abs/1207.3907.

[101] Danecek P, Auton A, Abecasis G, Albers CA, Banks E, DePristo MA, et al. The variant call format and VCFtools. Bioinformatics. 2011 Aug 1;27(15):2156–8. Available from: https://academic.oup.com/bioinformatics/article/27/15/2156/402296. doi: 10.1093/bioinformatics/btr330 21653522 PMC3137218

[102] Wick R. rrwick/Filtlong: quality filtering tool for long reads. GitHub. 2017. Available from: https://github.com/rrwick/Filtlong.

[103] Sedlazeck FJ, Rescheneder P, Smolka M, Fang H, Nattestad M, Von Haeseler A, et al. Accurate detection of complex structural variations using single-molecule sequencing. Nature Methods. 2018;15(6):461–8. Available from: 10.1038/s41592-018-0001-7. 29713083 PMC5990442

[104] Clark TA, Murray IA, Morgan RD, Kislyuk AO, Spittle KE, Boitano M, et al. Characterization of DNA methyltransferase specificities using single-molecule, real-time DNA sequencing. Nucleic Acids Research. 2012;40(4). Available from: doi: 10.1093/nar/gkr1146 22156058 PMC3287169

[105] Wick RR, Judd LM, Cerdeira LT, Hawkey J, Méric G, Vezina B, et al. Trycycler: consensus long-read assemblies for bacterial genomes. Genome Biology 2021 22:1. 2021 Sep 14;22(1):1–17. Available from: https://genomebiology.biomedcentral.com/articles/10.1186/s13059-021-02483-z. 34521459 10.1186/s13059-021-02483-z PMC8442456

[106] Kolmogorov M, Yuan J, Lin Y, Pevzner PA. Assembly of long, error-prone reads using repeat graphs. Nature Biotechnology. 2019 Apr 1;37(5):540–6. Available from: https://www.nature.com/articles/s41587-019-0072-8. doi: 10.1038/s41587-019-0072-8 30936562

[107] Wang H, Marcišauskas S, Sánchez BJ, Domenzain I, Hermansson D, Agren R, et al. RAVEN 2.0: A versatile toolbox for metabolic network reconstruction and a case study on *Streptomyces coelicolor*. PLoS Computational Biology. 2018 Oct 1;14(10):e1006541. Available from: https://journals.plos.org/ploscompbiol/article?id=10.1371/journal.pcbi.1006541. 30335785 10.1371/journal.pcbi.1006541 PMC6207324

[108] Ruan J, Li H. Fast and accurate long-read assembly with wtdbg2. Nature Methods. 2020 Dec 9;17(2):155–8. Available from: https://www.nature.com/articles/s41592-019-0669-3. doi: 10.1038/s41592-019-0669-3 31819265 PMC7004874

[109] Li H. Minimap2: pairwise alignment for nucleotide sequences. Bioinformatics. 2018 Sep 15;34(18):3094–100. Available from: https://academic.oup.com/bioinformatics/article/34/18/3094/4994778. doi: 10.1093/bioinformatics/bty191 29750242 PMC6137996

[110] Li H. Minimap and miniasm: Fast mapping and de novo assembly for noisy long sequences. Bioinformatics. 2016 Jul 15;32(14):2103–10. Available from: https://academic.oup.com/bioinformatics/article/32/14/2103/1742895. doi: 10.1093/bioinformatics/btw152 27153593 PMC4937194

[111] Walker BJ, Abeel T, Shea T, Priest M, Abouelliel A, Sakthikumar S, et al. Pilon: An Integrated Tool for Comprehensive Microbial Variant Detection and Genome Assembly Improvement. PLOS ONE. 2014 Nov 19;9(11):e112963. Available from: https://journals.plos.org/plosone/article?id=10.1371/journal.pone.0112963 25409509 10.1371/journal.pone.0112963 PMC4237348

[112] Seemann T. Prokka: Rapid prokaryotic genome annotation. Bioinformatics. 2014 Jul 15;30(14):2068–9. Available from: https://pubmed.ncbi.nlm.nih.gov/24642063/. doi: 10.1093/bioinformatics/btu153 24642063

[113] Seemann T. GitHub—tseemann/barrnap: Bacterial ribosomal RNA predictor. GitHub. 2019. Available from: https://github.com/tseemann/barrnap.

[114] Laslett D, Canback B. ARAGORN, a program to detect tRNA genes and tmRNA genes in nucleotide sequences. Nucleic Acids Research. 2004;32(1):11–6. Available from: /pmc/articles/PMC373265/. doi: 10.1093/nar/gkh152 14704338 PMC373265

[115] Matthey N, Stutzmann S, Stoudmann C, Guex N, Iseli C, Blokesch M. Neighbor predation linked to natural competence fosters the transfer of large genomic regions in *Vibrio cholerae*. Elife. 2019 Sep 1;8. doi: 10.7554/eLife.48212 31478834 PMC6783263

[116] Li H. A statistical framework for SNP calling, mutation discovery, association mapping and population genetical parameter estimation from sequencing data. Bioinformatics. 2011 Nov 1;27(21):2987–93. Available from: https://academic.oup.com/bioinformatics/article/27/21/2987/217423. doi: 10.1093/bioinformatics/btr509 21903627 PMC3198575

[117] Kumar S, Stecher G, Li M, Knyaz C, Tamura K. MEGA X: Molecular evolutionary genetics analysis across computing platforms. Molecular Biology and Evolution. 2018 Jun 1;35(6):1547–9. Available from: /pmc/articles/PMC5967553/ doi: 10.1093/molbev/msy096 29722887 PMC5967553

[118] Keller LE, Rueff ASS, Kurushima J, Veening JWW. Three new integration vectors and fluorescent proteins for use in the opportunistic human pathogen *Streptococcus pneumoniae*. Genes (Basel). 2019 May 22;10(5):394. Available from: https://www.mdpi.com/2073-4425/10/5/394/htm. doi: 10.3390/genes10050394 31121970 PMC6562690

[119] Lee MS, Seok C, Morrison DA. Insertion-duplication mutagenesis in *Streptococcus pneumoniae*: Targeting fragment length is a critical parameter in use as a random insertion tool. Applied and Environmental Microbiology. 1998;64(12):4796–802. Available from: https://journals.asm.org/doi/abs/10.1128/AEM.64.12.4796-4802.1998. 9835564 10.1128/aem.64.12.4796-4802.1998 PMC90924

[120] Madeira F, Park Y mi, Lee J, Buso N, Gur T, Madhusoodanan N, et al. The EMBL-EBI search and sequence analysis tools APIs in 2019. Nucleic Acids Research. 2019 Jul 2;47(W1):W636–41. Available from: https://academic.oup.com/nar/article/47/W1/W636/5446251. doi: 10.1093/nar/gkz268 30976793 PMC6602479

[121] Edgar RC. MUSCLE: Multiple sequence alignment with high accuracy and high throughput. Nucleic Acids Research. 2004;32(5):1792–7. Available from: https://pubmed.ncbi.nlm.nih.gov/15034147/. doi: 10.1093/nar/gkh340 15034147 PMC390337

[122] Price MN, Dehal PS, Arkin AP. Fasttree: Computing large minimum evolution trees with profiles instead of a distance matrix. Molecular Biology and Evolution. 2009 Jul 1;26(7):1641–50. Available from: https://academic.oup.com/mbe/article/26/7/1641/1128976. doi: 10.1093/molbev/msp077 19377059 PMC2693737

[123] Letunic I, Bork P. Interactive tree of life (iTOL) v5: An online tool for phylogenetic tree display and annotation. Nucleic Acids Research. 2021 Jul 2;49(W1):W293–6. Available from: https://academic.oup.com/nar/article/49/W1/W293/6246398. doi: 10.1093/nar/gkab301 33885785 PMC8265157

[124] Tettelin H, Nelson KE, Paulsen IT, Eisen JA, Read TD, Peterson S, et al. Complete genome sequence of a virulent isolate of *Streptococcus pneumoniae*. Science (1979). 2001 Jul 20;293(5529):498–506. Available from: https://pubmed.ncbi.nlm.nih.gov/11463916/ *Streptococcus pneumoniae*. doi: 10.1126/science.1061217 11463916

