# Supplementary figures and images for: The acquisition of clinically relevant amoxicillin resistance in Streptococcus pneumoniae requires ordered horizontal gene transfer of four loci
Source: PLoS Pathog. 2022 Jul 25;18(7):e1010727. doi: 10.1371/journal.ppat.1010727 (PMC9352194; doi:10.1371/journal.ppat.1010727)

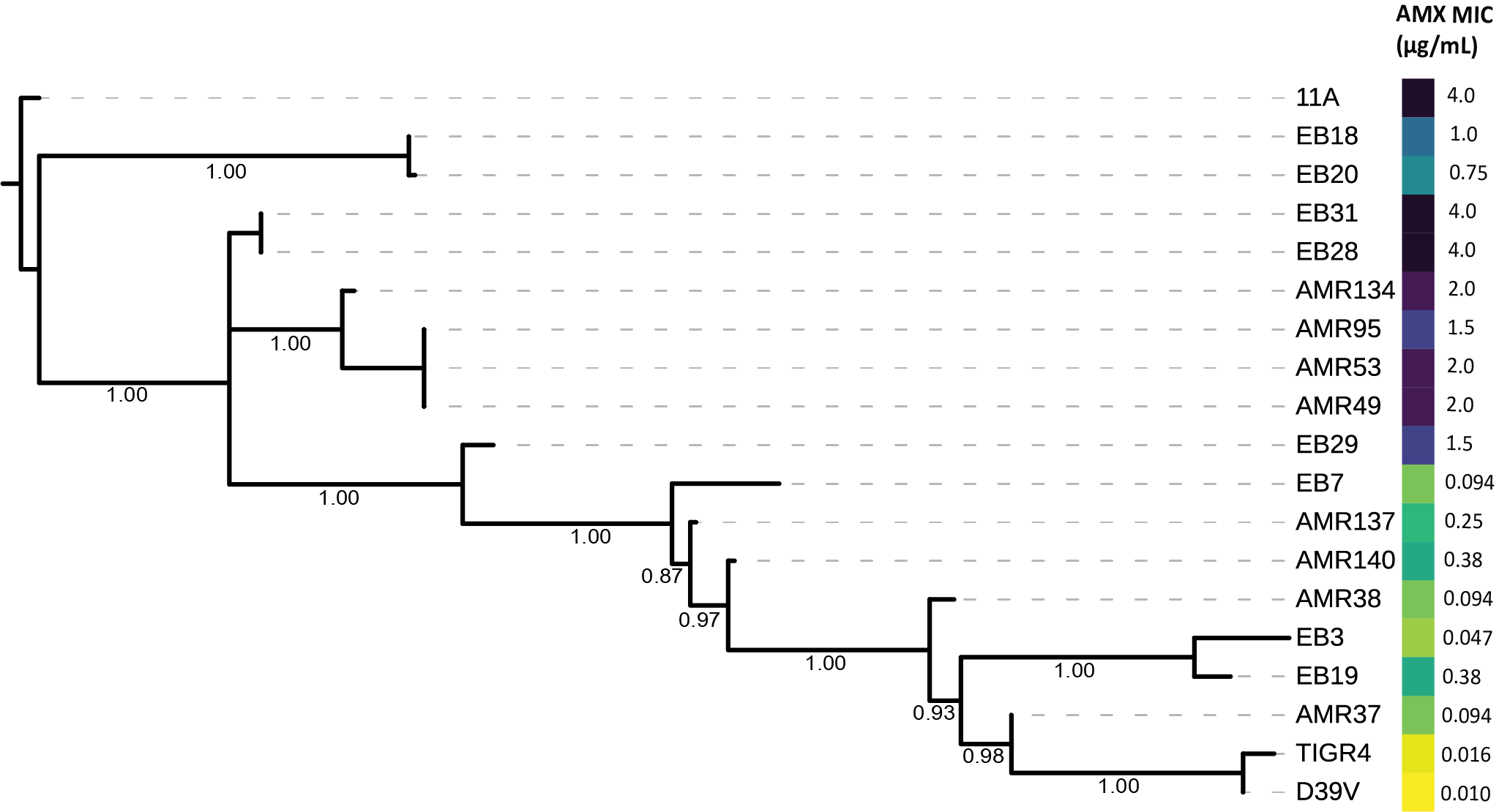

Supplement: S1 Fig — (A) Bocillin-FL labelled cell extracts imaged in an Amersham Typhoon with Cy2 filter setup showing PBP binding affinities, from Fig 3B Coomassie stain of Bocillin-FL gel from Fig 3C Bocillin-FL labelled cell extracts imaged in an Amersham Typhoon Cy2 filter setup from Fig 8D Coomassie stain of Bocillin-FL gel from Fig 8. (TIF) [file ppat.1010727.s009.tif]

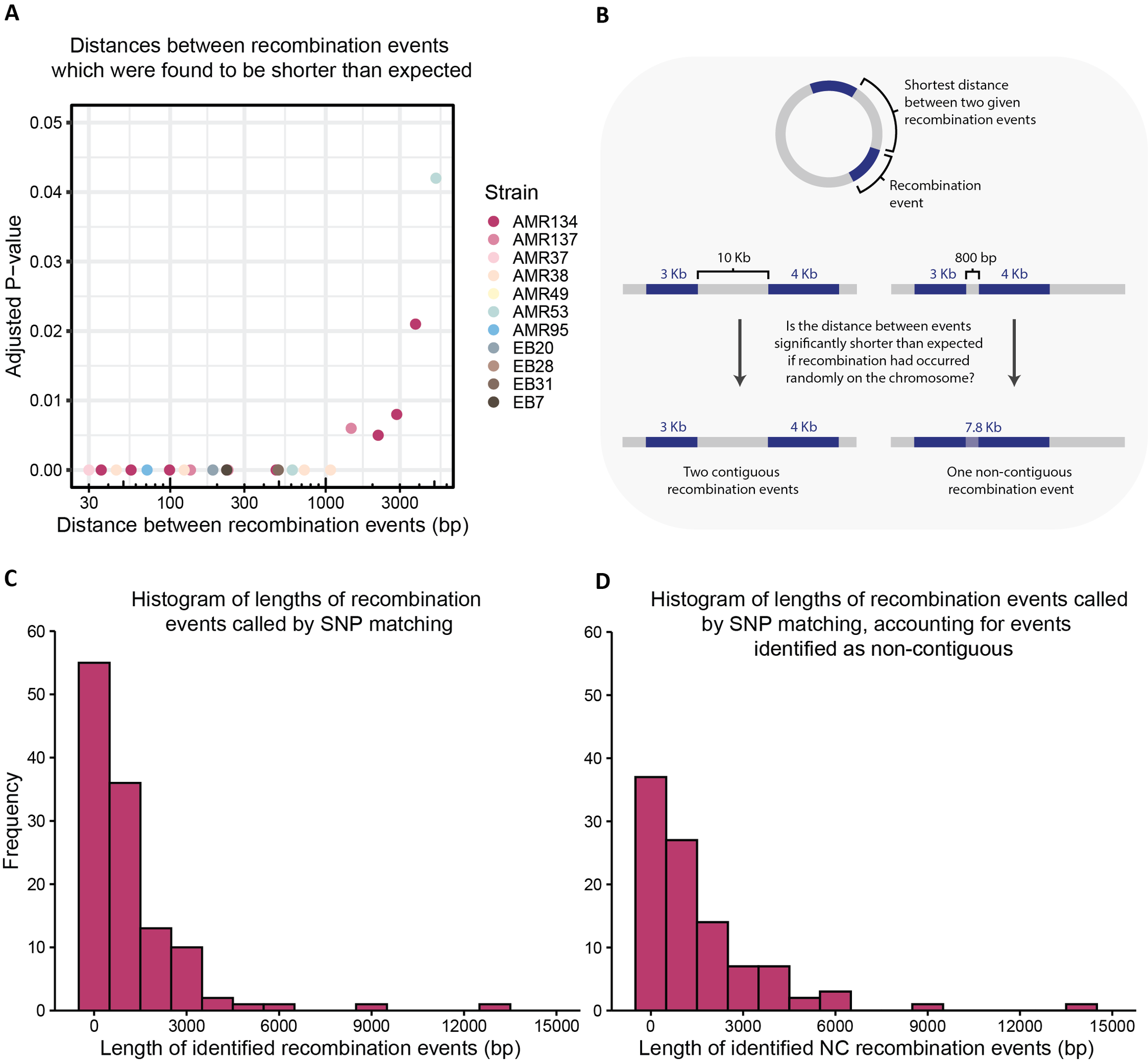

Supplement: S2 Fig — (A) Distances between recombination events which were found to shorter than expected under a null hypothesis where events occur randomly around the chromosome. (B) Schematic showing how events were treated if they were found to be closer to each other than expected. (C) Histogram of recombination events length across all sequenced recombinant strains if non-contiguous recombination is not taken into account. (D) Histogram of recombination events length across all sequenced recombinant strains if non-contiguous recombination is accounted for. (TIF) [file ppat.1010727.s010.tif]

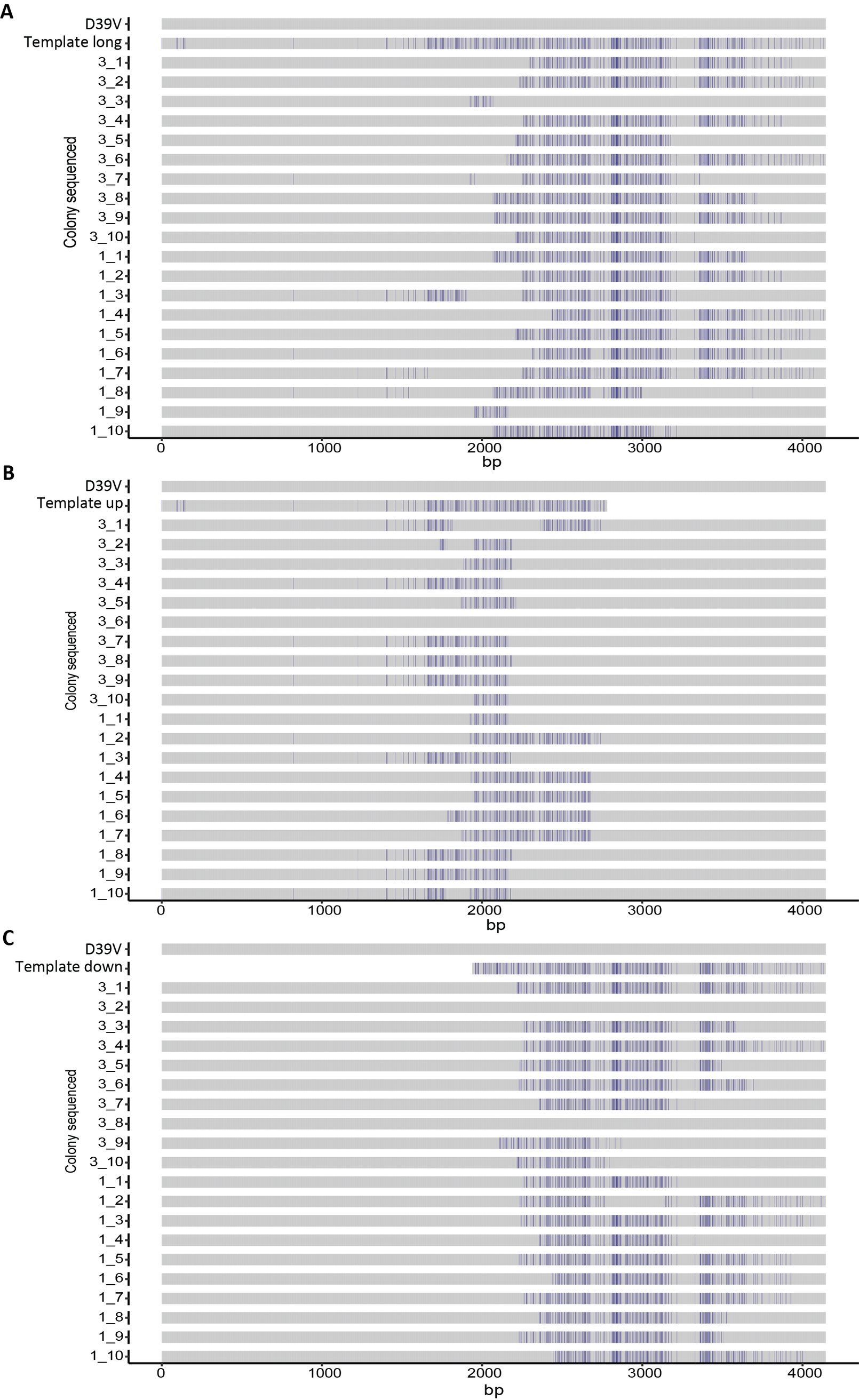

Supplement: S3 Fig — Facets show colonies selected from different donor templates which correspond to Fig 6. Bases which match the recipient (D39V) are coloured grey, those which match the donor (11A) are shown in blue. (A) Long, (B) Up, (C) Down. (TIF) [file ppat.1010727.s011.tif]

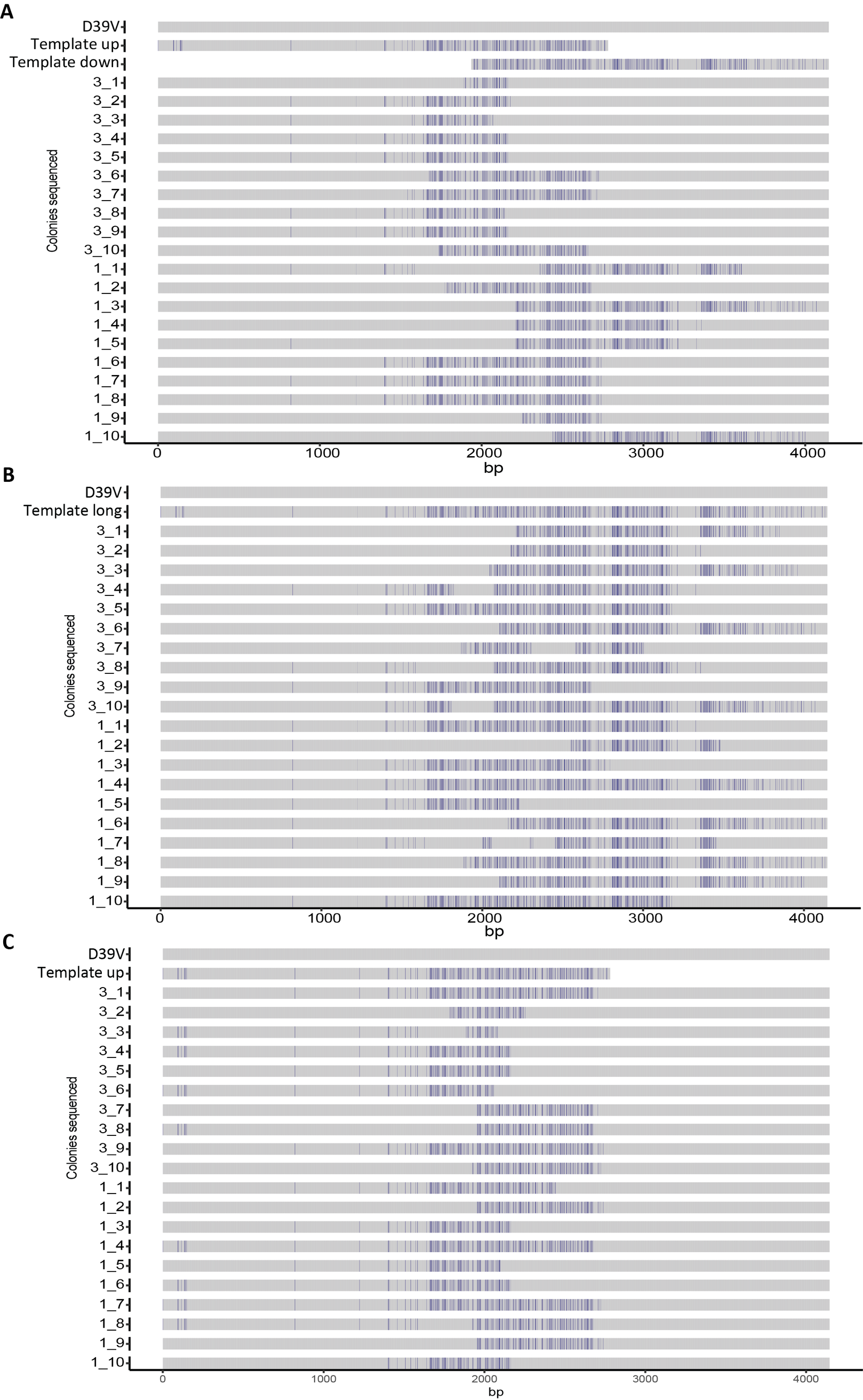

Supplement: S4 Fig — Facets show colonies selected from different donor templates which correspond to Fig 6. Bases which match the recipient (D39V) are coloured grey, those which match the donor (11A) are shown in blue. (A) Up & Down, (B) Long-NH, (C) NH-Up. (TIF) [file ppat.1010727.s012.tif]

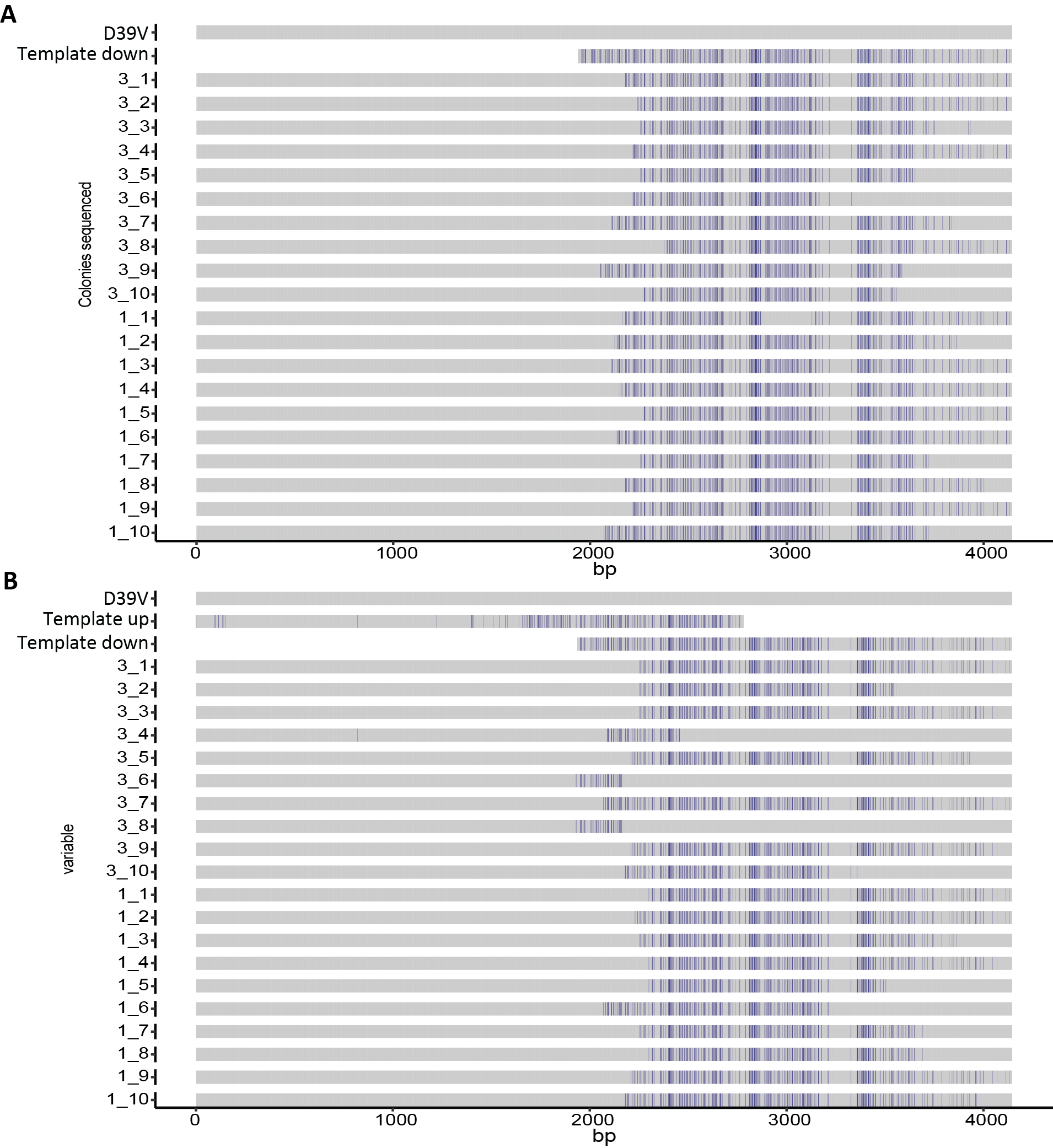

Supplement: S5 Fig — Facets show colonies selected from different donor templates which correspond to Fig 6. Bases which match the recipient (D39V) are coloured grey, those which match the donor (11A) are shown in blue. (A) Down-NH, and (B) NH-Up and Down-NH. (TIF) [file ppat.1010727.s013.tif]
